# Bioactive injectable mucoadhesive thermosensitive natural polymeric hydrogels for oral bone and periodontal regeneration

**DOI:** 10.3389/fbioe.2024.1384326

**Published:** 2024-05-28

**Authors:** Mohammad El-Nablaway, Fatema Rashed, Ehab S. Taher, Gamal A. Atia, Tarek Foda, Nourelhuda A. Mohammed, Ahmed Abdeen, Mohamed Abdo, Ioana Hînda, Ana-Maria Imbrea, Noha Taymour, Ateya M. Ibrahim, Ahmed M. Atwa, Samah F. Ibrahim, Mahmoud M. Ramadan, Stefania Dinu

**Affiliations:** ^1^ Department of Medical Biochemistry, Faculty of Medicine, Mansoura University, Mansoura, Egypt; ^2^ Department of Basic Medical Sciences, College of Medicine, AlMaarefa University, Riyadh, Saudi Arabia; ^3^ Department of Basic Medical and Dental Sciences, Faculty of Dentistry, Zarqa University, Zarqa, Jordan; ^4^ Department of Oral Medicine, Periodontology, and Diagnosis, Faculty of Dentistry, Suez Canal University, Ismailia, Egypt; ^5^ Oral Health Sciences Department, Temple University’s Kornberg School of Dentistry, Philadelphia, PA, United States; ^6^ Physiology and Biochemistry Department, Faculty of Medicine, Mutah University, Al Karak, Jordan; ^7^ Department of Forensic Medicine and Toxicology, Faculty of Veterinary Medicine, Benha University, Toukh, Egypt; ^8^ Department of Animal Histology and Anatomy, School of Veterinary Medicine, Badr University in Cairo (BUC), Cairo, Egypt; ^9^ Department of Biology, Faculty of Agriculture, University of Life Sciences “King Michael I” from Timișoara, Timișoara, Romania; ^10^ Department of Biotechnology, Faculty of Bioengineering of Animal Resources, University of Life Sciences “King Mihai I” from Timisoara, Timișoara, Romania; ^11^ Department of Substitutive Dental Sciences, College of Dentistry, Imam Abdulrahman bin Faisal University, Dammam, Saudi Arabia; ^12^ Department of Administration and Nursing Education, College of Nursing, Prince Sattam bin Abdulaziz University, Al-Kharj, Saudi Arabia; ^13^ Department of Family and Community Health Nursing, Faculty of Nursing, Port-Said University, Port Said, Egypt; ^14^ Department of Pharmacology and Toxicology, Faculty of Pharmacy, Egyptian Russian University, Cairo, Egypt; ^15^ Department of Internal Medicine, College of Medicine, Princess Nourah bint Abdulrahman University, Riyadh, Saudi Arabia; ^16^ Department of Clinical Sciences, College of Medicine, University of Sharjah, Sharjah, United Arab Emirates; ^17^ Department of Pedodontics, Faculty of Dental Medicine, Victor Babes, University of Medicine and Pharmacy Timisoara, Timisoara, Romania; ^18^ Pediatric Dentistry Research Center, Faculty of Dental Medicine, Victor Babes University of Medicine and Pharmacy Timisoara, Timisoara, Romania

**Keywords:** biomaterials, biopolymers, hydrogels, drug delivery, periodontitis, tissue engineering

## Abstract

Periodontitis is an inflammation-related condition, caused by an infectious microbiome and host defense that causes damage to periodontium. The natural processes of the mouth, like saliva production and eating, significantly diminish therapeutic medication residency in the region of periodontal disease. Furthermore, the complexity and diversity of pathological mechanisms make successful periodontitis treatment challenging. As a result, developing enhanced local drug delivery technologies and logical therapy procedures provides the foundation for effective periodontitis treatment. Being biocompatible, biodegradable, and easily administered to the periodontal tissues, hydrogels have sparked substantial an intense curiosity in the discipline of periodontal therapy. The primary objective of hydrogel research has changed in recent years to intelligent thermosensitive hydrogels, that involve local adjustable sol-gel transformations and regulate medication release in reaction to temperature, we present a thorough introduction to the creation and efficient construction of new intelligent thermosensitive hydrogels for periodontal regeneration. We also address cutting-edge smart hydrogel treatment options based on periodontitis pathophysiology. Furthermore, the problems and prospective study objectives are reviewed, with a focus on establishing effective hydrogel delivery methods and prospective clinical applications.

## 1 Introduction

Periodontitis is a persistent inflammation that negatively impacts tissues that support the teeth, resulting in periodontal tissue damage and potentially tooth loss. The clinical appearance ranges from traditional symptoms of inflammatory including pain, edema, and impairment of functionality, to total tooth loss. The teeth loosened at first, and tooth loss could take place later on. Periodontitis is caused by a disturbance in the oral microbiota’s equilibrium and host defenses (dysbiosis), which has been connected to a variety of systemic illnesses. In adults, dysbiosis could be the reason for periodontitis, which might result in inflammatory alterations in the bone and connective tissue (Jensen et al., 2018). Periodontitis is among the most significant chronic illnesses in the world, impacting more than 11% of people around the globe. It has been estimated that between 800 million and 1.4 billion individuals worldwide are affected by severe periodontitis. The fundamental etiology of periodontitis is bacterial plaque imbalances and host immunological reactions which cause the early inflammation (Könönen et al., 2019b).

To lower the pathogenic risk of periodontitis, first-line therapy should be performed. Furthermore, clinical therapy of periodontitis necessitates the use of adjuvant medications to inhibit the proliferation of harmful bacteria, as well as decrease the inflammatory conditions. Metronidazole, penicillin, minocycline, doxycycline, and other antibiotics are used to treat periodontitis. Reaching therapeutic efficacy of drugs requires accumulation in periodontal tissues, on the contrary, needs an increased level of systemic administration of antibiotics, which could end up in bacterial imbalances. Resistance to medication, and a likelihood of liver/kidney damage (Prakasam et al., 2012; Toledano et al., 2021).

Injectable thermos-responsive hydrogels have gained popularity as potential biomaterials for usage in tissue engineering during the last decade because they are typically cytocompatible, biodegradable, and may resemble the architecture of the extracellular matrix (ECM). With the growing rise of cell-based treatments, there is an increasing desire to produce injectable hydrogels as cellular transporters, eliminating open surgery and pushing the utilization of less hazardous materials and cellular transportation methods. The injectable hydrogels are naturally flexible, allowing them to fill lesions of varying sizes, and a minimally invasive method aids in the administration of biologically active substances and cells (Chen et al., 2023; Hasanzadeh et al., 2023).

## 2 Categorization of hydrogels

Hydrogels are categorized into numerous types according to the polymeric, and polyelectrolytic frameworks that are employed. They are classed as homo-, co-, or interpenetrating polymeric hydrogels based on the manufacturing technique. They might be intelligent or ordinary hydrogels based on their chemical makeup. It is feasible to differentiate between organic, semi-synthetic, and artificial hydrogel forms (Atia et al., 2023a). Nonionic, anionic, cationic, and ampholytic hydrogels may additionally be classed according to their charges. According to their biological decomposition, they are classed as biodegradable or non-biodegradable. They are characterized as physical, biological, or chemical hydrogels according to the kind of cross linker used.

Environmental variables can cause physical hydrogels to transform from solution to gel form. Chemical hydrogels are covalently bonded polymers that exceed other materials in terms of physical durability and ability to withstand deterioration. Biological substances such as amino acids, enzymes, and so on are employed in the gelation process of biochemical hydrogels (Atia et al., 2023b).

### 2.1 Based on the source

**FIGURE 1:**
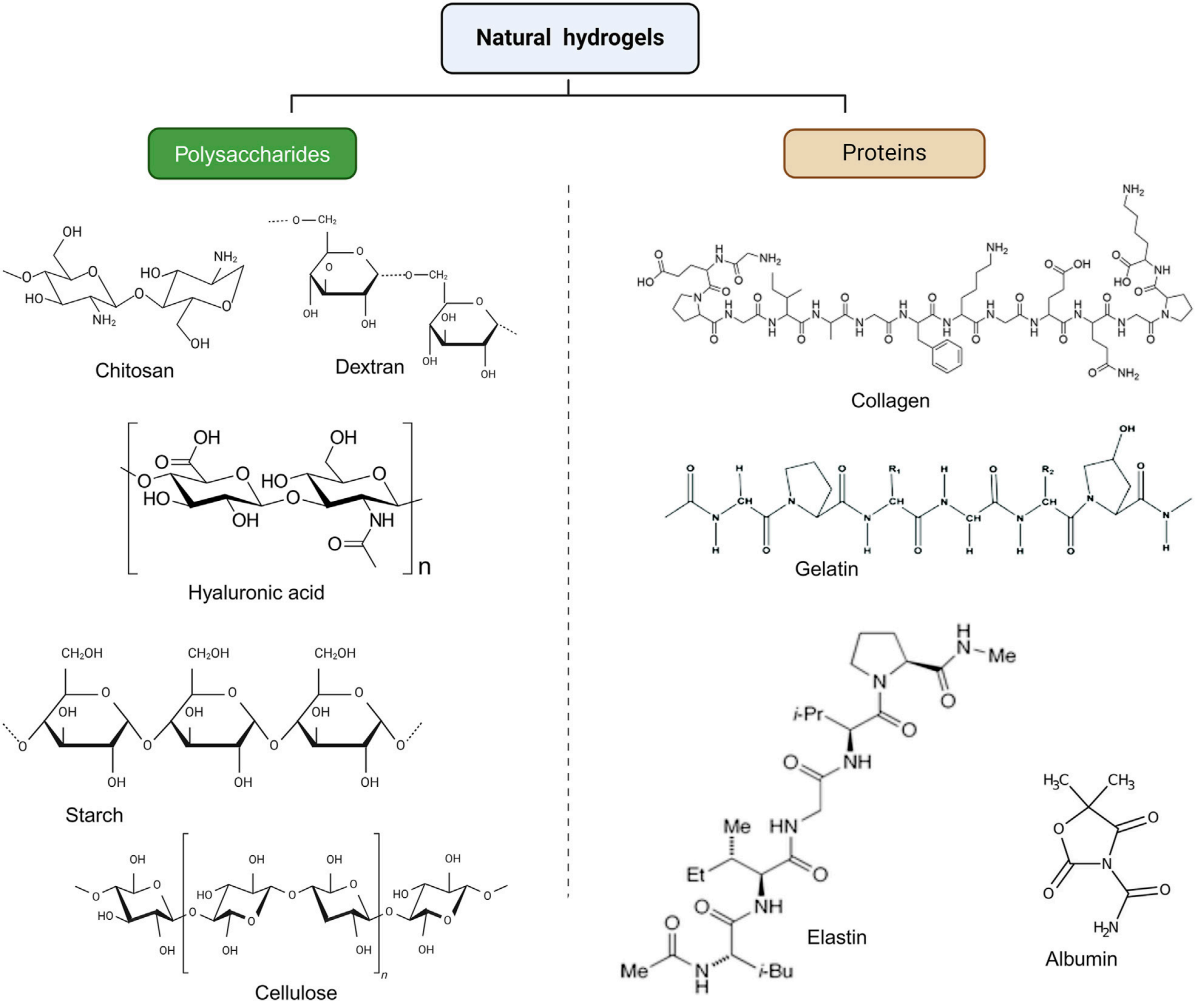
Classification of hydrogels according to origin.

They are categorized as organic, artificial, or semi-synthetic based on their sources (Figure 1). Most artificial hydrogels are produced *via* the conventional polymerization of vinyl or vinyl-activated monomers. The ideal expansion characteristics of these artificial hydrogels depend on the monomer hydrophilic characteristics and the degree of cross-linking. A monomer with double functionalities is routinely used to carry out the local crosslinking process. Natural hydrogels are made up of polymers found in nature (Atia et al., 2023a).

### 2.2 Based on the durability of hydrogels

Hydrogels can be either durable or biodegradable depending on the way they respond in physiological settings. A large body of recent studies have concentrated on the advancement and practical usage of new biodegradable hydrogels. They are now engaged in numerous distinct industries, including biological applications (Alkhursani et al., 2022a). The chain scission of the degradable components inside the hydrogel matrix produces minimal molecular mass oligomers. The oligomers formed are then either removed by the body or further degraded.

### 2.3 Based on interaction with environmental stimuli

**FIGURE 2:**
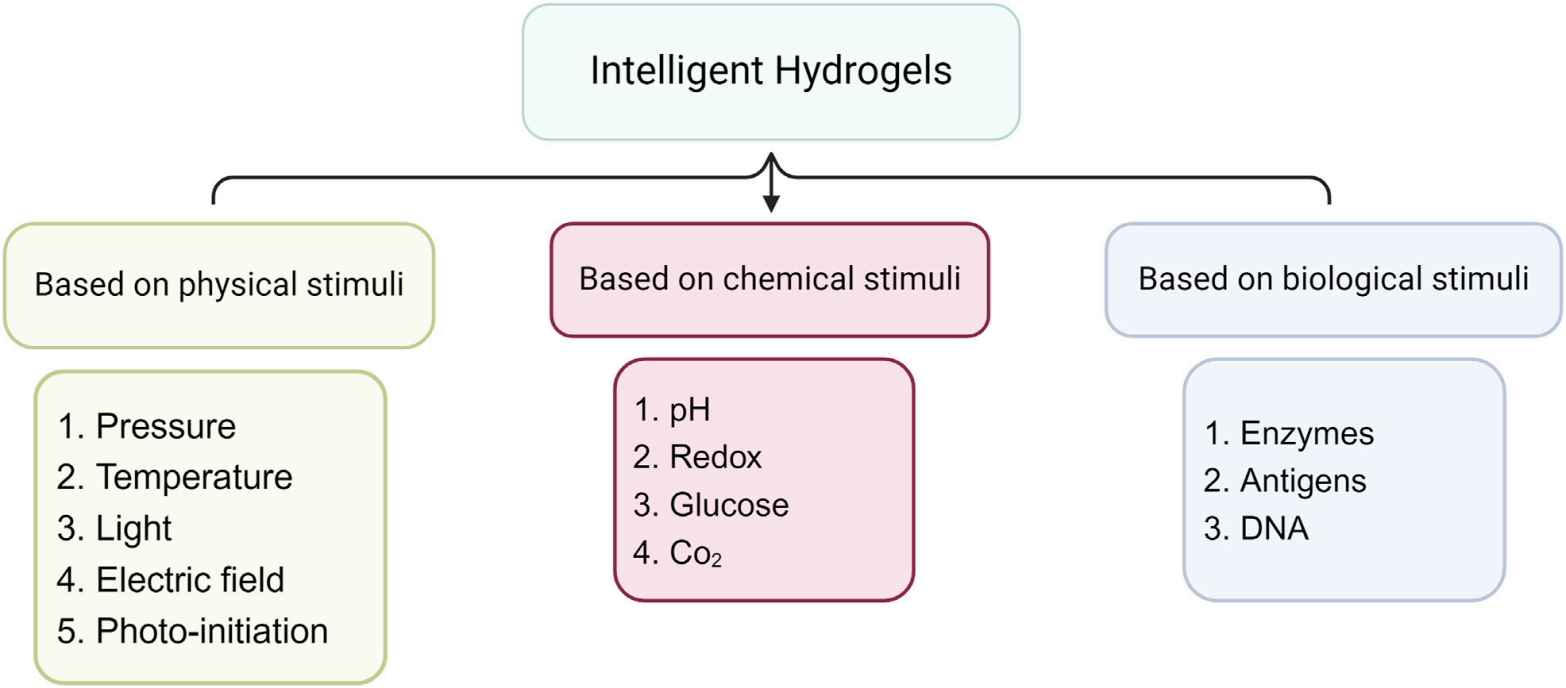
Stimuli-responsive hydrogels.

Over the last few years, major advancement was achieved in the synthesis and investigation of a novel class of hydrogels known as “smart hydrogels.” Despite similarities in preparation methods and methods of characterization for traditional forms, intelligent hydrogels may display uncommon variations in swelling patterns, framework architecture, and/or physical features as they react to several external factors like pH, temperature, or electricity (Atia et al., 2022a). The alterations that occur in intelligent hydrogels when exposed to any of these external factors generally evaporate when the stimulus is removed, and the hydrogels revert to their earlier state, and so on in a process that can be reversed (Figure 2).

### 2.4 Cross-linking techniques of hydrogels

Crosslinks, are required to prevent hydrophilic polymer chains/segments from dissolving in the aqueous phase. Rheology is another approach for describing hydrogels. Water-based solutions of hydrophilic polymers at mild or intermediate levels, with no significant chain entanglement, typically exhibit Newtonian behavior (Klouda, 2015). When crosslinks between polymer chains develop, the resulting networks exhibit viscoelastic and, in certain cases, pure elastic behavior. Considering their water-absorbing capacity, hydrogels are not only being studied by investigators who are studying the basic properties of swollen polymerized systems, but they also have discovered popular uses in numerous fields. As previously stated, crosslinks are required in a hydrogel to keep the hydrophilic polymers from dissolving in aqueous conditions (Huang et al., 2019). Hydrogels have been prepared using an extensive range of crosslinking processes. Labile bonds are routinely added into hydrogels because their biodegradability is helpful for numerous purposes. These linkages can be found in either the core of the polymer or the crosslinks employed to form the gel (Tanga et al., 2023). According to the specified architecture and the desired use, hydrogels could be physically, or chemically cross linked (Table 1).

**TABLE 1:** Difference between physical and chemical hydrogel.

Point of comparison	Physical hydrogel	Chemical hydrogel	References
Bonding type	Ionic, H-bonding, or hydrophobic interactions, as well as molecular interactions	Covalent interaction creates chemical hydrogels	Suflet et al. (2021); Li W et al. (2023)
Bonding nature	Physical hydrogels are regarded as reversed gels since the above linkages are weaker	As a result of the power of covalent bonding, these are referred to as persistent or unchangeable	Bustamante-Torres et al. (2021)
Cross linking	These are made without the utilization of chemical modifications or cross-linkers	These are generated via chemical alteration or cross-linking substances	Yang, (2022)
Physical properties	It exhibits uneven outcomes *in vivo* because it is rigid concerning parameters like gelation duration, gel pore dimensions, chemical customization, and breakdown or solubility	In terms of gelation time, porosity dimensions, chemical functionality, and breakdown or disintegration, it exhibits flexibility	Kaczmarek et al. (2020)
Degradation	They are less resistant to deterioration	They are particularly resilient to degradation	Xue et al. (2022)
Form	These are uniform	These lack homogeneity	Ahn et al. (2021)
Networks	The polymer framework of physical hydrogels has both hydrophilic and hydrophobic areas	Chemical hydrogels have zones with a higher cross-linking intensity than ordinary hydrogels	Yang, (2022)
Mechanical properties	Due to the reversible physical bonds, these hydrogels have weak mechanical characteristics	Compared to hydrogels that have been physically cross-linked, these hydrogels possess superior mechanical properties	Summonte et al. (2021)
Pharmaceuticals loading	These gels have significant promise for the inclusion of bioactive compounds	These could be engaged in distinct applications like pharmaceuticals, agricultural, food, and cosmetics industries	Lima et al. (2020); Paiva et al. (2024)

#### 2.4.1 Chemical crosslinking

Among the most widely used methods for creating hydrogels is the chemical crosslinking of hydrophilic polymers. In this method, a bi-functional crosslinker is incorporated into a diluted solution of a hydrophilic polymer, and the polymer requires an appropriate function to interact with the crosslinker. This approach may be engaged in developing hydrogels from both organic and manufactured hydrophilic polymers (Yamada et al., 1997).

Chemical cross-linked hydrogels are hydrogels that might be covalently bonded from fluid to solid state. This approach is also applied in situ hydrogel platforms. This approach uses a variety of processes to produce hydrogels, including optical polymerization, enzymatic processes, and click reactions (Xue et al., 2022). Chemically crosslinked hydrogels have been investigated due to their high durability (Ullah and Lim, 2022).

##### 2.4.1.1 Enzymatic reactions

Enzymatic reactions occur in an abundance of enzymes in an ecological setting. As a result, this approach has received a great deal of interest for cellular technologies. Enzymatic reactions necessitate appropriate cross-linking circumstances, which include physiological pH, biological surroundings, and temperature (Badali et al., 2021). A key benefit of this technology for hydrogel creation is the unique enzyme substrate, which possesses the capacity to inhibit the penetration of harmful chemicals induced by adverse responses. Horseradish peroxidase (HPR), modified glutamines, and tyrosinases are some of the enzymatic accelerators utilized to create hydrogel systems for tissue engineering. HPR is one of the enzymes utilized to generate hydrogels in this process because of its great mechanical stability and simplicity of filtration (Bae et al., 2015). As a result, it is employed in a variety of health-related purposes including the release of medications, rehabilitation and regeneration, and tissue engineering. The HPR-H_2_O_2_ enzyme water system is commonly used to produce natural hydrogels from chitosan, hyaluronic acid, dextran, and gelatin. In a study by Kurosawa et al. It was produced by enzymatic processes and utilized for releasing therapeutic proteins. In this investigation, hyaluronic acid has been modified with tyramine and labelled with the fluorescent marker amino fluorescein. The subcutaneous gelling time was shortened, indicating that the utilization of enzymatic processes to generate cross-linking is an appropriate approach for hydrogel creation owing to its versatility and cell friendliness (Kurisawa et al., 2005).

##### 2.4.1.2 Click reaction

Sharples et al. describe click chemistry as “specific forms of processes with the inclusion of an initiator that exhibit superior rapidity and efficacy, outstanding physiological characteristics, and favorable reactions parameters” (Nguyen et al., 2015; Guaresti et al., 2018). It performs a substantial part in polymer production and triggering, and it is an efficient and versatile approach for functionalizing compounds. Because of the advantages of click chemistry, it is utilized to produce hydrogels, nanogels, and microgels. It has been served as a foundation for tissue engineering and medication delivery. Click chemistry is seen as a promising platform for dynamic chemically cross-linked polysaccharide-based. Click reactions typically encompass an extensive number of processes, such as: copper (I) interactions (catalyzed by alkyne azide sequestered), catalytic reactions of free pairs of alkyne azides, silicosterone reaction with disaccharide (DA), among which the intergroup reactions Alkynes, and azides are the most prominent instances of click chemistry because of their benefits, including outstanding effectiveness in physiological settings and good specificity (Nguyen et al., 2015). In the lack of thermally reversible byproducts which enable the level of interaction to be regulated, the reaction occurs without a catalyst or primer, preserving the material’s biocompatibility (Guaresti, García–Astrain, Aguirresarobe, Eceiza and Gabilondo, 2018). Starch-based hydrogels were created via a click reactions between the thiol and allyl groups of starch for biomedical and tissue engineering purposes. The resultant hydrogel demonstrated remarkable swelling and biological degradation (Samadian et al., 2020).

#### 2.4.2 Physical crosslinking

Physical crosslinking interactions include polyelectrolyte complexation, hydrogen bonding, and hydrophobic association, and the hydrogels produced by this approach are typically synthesized under moderate circumstances.

A. *Polyelectrolyte complexation (Ionic interactions):* It involves the establishment of linkages between pairings of chargeable locations along the polymeric skeletons. The stabilities of the generated electrolytic linkages vary depending on the pH of the media (Anal and Stevens, 2005).
B. *Hydrogen bonding:* It can also play a role in hydrogel generation (Mozafari, 2007). A hydrogen bond is generated by combining an electron-deficient hydrogen atom with an electron-rich functioning group.
C. *Hydrophobic interaction:* Hydrophobic interactions are another approach for producing Hydrogels (Nakashima et al., 1977). Because of the low interfacial adhesion, the mechanical properties of these hydrophobically mixed polymers are often poor. However, this method of hydrogel synthesis offers several advantages, such as a cheap system cost.

## 3 Injectable thermosensitive hydrogels

Injectable hydrogels are potential tissue engineering platforms, thanks to their elevated number of water-like tissues, capacity to uniformly encapsulate cells, effective transport of mass, readily modified physical characteristics, and less invasive distribution (Sun et al., 2020). The injectable architecture of the hydrogels gives the appealing characteristic of easy and homogeneous cell dispersion prior to gelation within any defect size or form.

Thermo-responsive polymers are instances of “intelligent polymeric platforms” that respond to variations in temperature (Alkhursani et al., 2022b). Their temperature responsiveness has been used in a variety of biological applications. They often have a lower critical solution temperature (LCST) and may produce a gel reversibly when temperatures rise (Cook et al., 2021). Some have an upper critical solution temperature (UCST) above which they become soluble and form gels at lower temperatures (Aguilar and San Román, 2019). The source of thermresponsive polymers might be either natural or manufactured (Chatterjee et al., 2018). Thermo-sensitive hydrogels have become popular because of the controlled dissolution of temperature-sensitive bioactive compounds. Thermo-responsive hydrogels have various benefits as a means of transportation method. The hydrogel’s dependent upon temperature characteristics makes it different from conventional hydrogels, which require surgical implantation., permitting local administration and avoidance of first-pass metabolism.

The temperatures-thermo-responsive gel reacts to temperature since no denaturing cross-linking agent is used, and the temperature-triggered sol-gel transformation is less harmful and more appropriate to be injected. Encapsulation in a streaming state ensures that medicinal materials are distributed uniformly in hydrogels, whereas fast sol-to-gel transformation at physiologic temperature minimizes the premature bursting discharge of pharmaceuticals, instead enabling sustained release behavior. Ultimately, the flowable delivery state allows the hydrogel shape to be controlled.

## 4 Requirements of injectable thermosensitive hydrogels for periodontal regeneration

**FIGURE 3:**
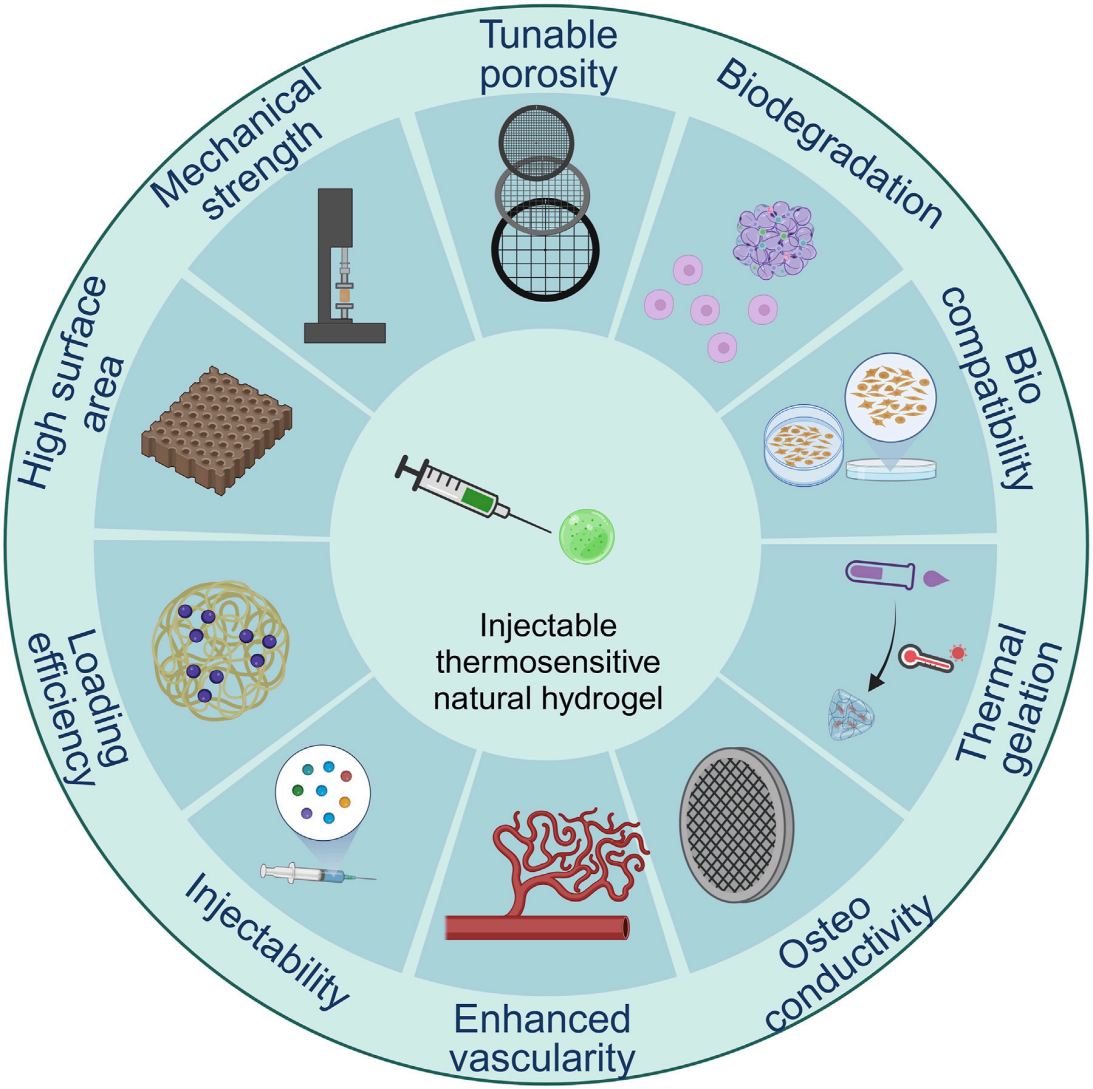
Design criteria of injectable thermos-sensitive hydrogels.

The architectural specifications should also incorporate traditional biomechanical metrics (like being biodegradable, porous, and has adequate surface chemistry), as well as biological efficiency measures (including being biocompatible and enabling cellular attachment), as well as evidence of improved angiogenesis (Figure 3). Furthermore, while creating hydrogel scaffolds for tissue engineering, availability as well as economic viability should be considered.

A solution that flows easily at standard ambient temperature and gels following injection into the body is the ideal option (Madan et al., 2009) The molecular makeup of thermos-responsive polymers is characterized by a balanced arrangement of hydrophobic and hydrophilic links. To maintain this condition of equilibrium, even little temperature changes in the watery polymer solution may result in a recalculation of the hydrophobic and hydrophilic relationships between the polymeric subunits and aqueous particles (Matanović et al., 2014). When the concentration rises, the transition temperature falls, and the curve shifts to the left once more (Ahmed, 2015). Many techniques, including spectrometry, differential scanning calorimetry, and rheology, can be utilized to evaluate the thermos-responsiveness of hydrogels.

### 4.1 Mechanisms of gelation

Hydrophobic groups like methyl, ethyl, and propyl are frequent properties of thermosensitive hydrophilic homopolymers (Haq et al., 2017). Inter-polymer, polymer-water, and water-water interactions occur in an aqueous polymer solution (Rivas et al., 2003). As previously stated, polymers are soluble below the LCST and eventually become non-polar above the LCST, culminating in the hydrogel. Because of polymer chain breakage and aggregation, light dispersion in solution increases with LCST. As a result, at the cloud point, two phases emerge: collapsed gel and water ejected from the gel. Temperature-dependent hydrogen (water-water) and hydrophobic reactions control the sol-gel transformation (Kitazawa et al., 2018). The solvated polymers rapidly dehydrate and convert to a more hydro-phobic architecture at the sol-gel transition point.

### 4.2 Biodegradation

A major criterion of scaffoldings for tissue engineering is that they maintain the multiplication of cells and desirable cell dispersion during the scaffold’s expected life. In several circumstances, the scaffolding`s life could be till full decay. As a result, the pace and amount of biodegradability are essential designation factors for tissue engineering hydrogels. Scaffolds that are semi-persistent or durable can be the best option for replacing the fundamental function of lost or injured tissue in various types of tissues. Degradable hydrogel scaffolds are frequently made by including cleavable bonds and/or cleavable components in the polymer skeleton. Physiological processes, notably enzyme metabolism, are used to degrade biodegradable hydrogel scaffolds (Ratner et al., 2004).

### 4.3 Biocompatibility

A material’s ability to comply with a suitable response from the host in any particular function is a crucial designing element in engineered tissue constructs,” according to the definition (Atia et al., 2022a). Some components utilized in hydrogel synthesis might be harmful if they seep into implanted cells or tissues. Irgacure, a routinely employed free radical photo-catalyst, has been shown to reduce cell survival even at low doses (Bryant et al., 2000). As a result, before usage, hydrogels generated for tissue engineering need normally be free of unprocessed toxic compounds. This purification can be achieved by using distinct approaches, such as dialysis and thorough solvent washing. Purification of hydrogel scaffolds can be difficult, if not impossible, in some situations, such as in the case of hydrogels generated using local gelation. As a result, while employing these gelation processes, special care should be taken to verify that all chemicals are non-toxic and sufficiently safe.

### 4.4 Pore dimensions and porosity distribution

A significant amount of linked porosity promotes cellular ingrowth, homogeneous cell distribution, and matrix neovascularization (y Leon, 1998). Not only is the level of permeability significant but so are numerous additional variables including porosity dimensions, volume, the distribution of pores, wall texture, and pore interconnection when developing a hydrogel scaffold for tissue engineering purposes. Pore interconnection, for example, is crucial for guaranteeing all cells are within 200 µm of the blood supply to be able to allow for the exchange of nutritional factors (Yang et al., 2001; Salgado et al., 2004).

Porosity dimensions are also a significant metric since pore blockage by cells would occur if the generated pores were too tiny, preventing Infiltration of cells, ECM synthesis, and neovascularization of the scaffold’s interior sections. Several new investigations have provided information on the influence the influence of scaffolding permeability on tissue regeneration, as well as the ideal hole sizes for various uses. For example, it has been established that the optimal pores dimension for neovascularity is 5 μm, and 200–350 µm for osteo-conductivity (Whang et al., 1999; El-Sherbiny and Yacoub, 2013).

### 4.5 Physical properties

The physical features of hydrogels as frameworks for tissue engineering might possess a crucial influence on linked or enclosed cells. After *in situ* gelation, hydrogels ought to exhibit sufficient mechanical strength to withstand biomechanical stress and provide short-term nourishment for cells (Hou, Paul, et al., 2004). Hydrogel stiffness, for example, has been discovered to influence stem cell growth. The crosslinking density is one of the main factors governing the mechanical conformance of hydrogel scaffolds; it can also have an impact on cells enclosed in hydrogel frameworks.

Compared to other scaffolds, injectable hydrogels are softer and more elastic because of their thermodynamic biocompatibility with water. Many parameters impact the durability of injectable hydrogels, including biomaterial nature, quantity, manufacturing technique, porosity, and crosslinking density. Physical hydrogels are often weaker than chemical ones. The inclusion of other groups into the polymeric framework enhances the mechanical features of injectable hydrogels (Chang et al., 2017; Atia et al., 2022a).

### 4.6 Persistence duration

A variety of parameters influence the duration or housing duration of thermos-responsive hydrogels, particularly the kind of arrangement of the monomer, the extent of polymer cross-linkage, the rate of polymer decomposition, and the strength of the thermal stimulation. It is crucial in determining the outcome of medication delivery.

Because plain thermosensitive polymers possess little durability, they may deteriorate in biological fluids, restricting the long-term release of encapsulated medicines. These polymers can be combined with strong mechanical properties polymers. As a result, the produced copolymers will possess a longer staying duration since their breakdown rates will be reduced, perhaps that results in an extended stay of the medication, eventually boosting effectiveness (Pardeshi et al., 2022).

### 4.7 Vascularity

Vascularization is required to offer an environment for the transfer of nutrients and the perfusion-based elimination of undesirable substances. Nevertheless, developing suitable scaffolds that enable and stimulate the growth of neovascularization is a challenging task (Mazloomnejad et al., 2023). Hydrogels have proven to be effective as vascularization promotion scaffolds in some tissue engineering applications. There are two basic techniques for encouraging the vascularization of a tissue-created scaffold (Wang et al., 2022). The first strategy incorporates growth factors into the hydrogel matrix to encourage angiogenesis from adjacent tissues to grow into the framework. The second method is to seed the framework with endothelial cells (ECs) (Shan and Wu, 2024).

Another strategy being researched for stimulating the vascularization of tissue engineering scaffolds is the recruitment of endothelial progenitor cells (EPCs). Yet, depending on EPCs and the natural adjacent vessels to penetrate the inserted platforms is a time-consuming procedure that needs considerable amounts of flowing EPCs (Peters, 2018). Because cells are not able to live more than a few hundred micrometers away from blood vessels, different kinds of cells inserted within the platform could succumb to necrosis while awaiting microvascular expansion. Furthermore, while EPCs constitute an infinite origin of cells for *in vitro* revascularization, their differentiation must be regulated (Gerecht et al., 2007).

### 4.8 Drug-delivery methods

It is mandatory to comprehend the transport approaches in the gelled matrix throughout the creation phase of a novel delivery system (Ćorković et al., 2021). A sustained release is envisaged if an injectable fluid containing a thermos-sensitive platform is injected into the body to alter its molecular makeup from sol to gel (Bellotti et al., 2021). Drug-release processes from hydrogels are categorized into four types: (1) diffusion, (2) erosion, (3) swelling, and (4) chemical control (Li and Mooney, 2016). Reservoir or matrix platforms are methods of drug administration with diffusion-controlled drug release (Qureshi et al., 2021). Drug diffusion out of a hydrogel is mostly determined by the Mesh dimensions inside the gel matrix, but it is also affected by the drug molecule’s hydrodynamic radius (Vigata et al., 2020).

Average mesh sizes described for swollen hydrogels vary from 5 to 100 nm, making them bigger than most small-molecule medicines (Atia et al., 2023a). As a result, tiny medications have a short duration of action, but macromolecules including oligonucleotides and protein drugs possess a longer duration of action (Agrahari et al., 2016). Erosion or swelling will be the primary mechanism for drug release when the hydrodynamic radius of the drug molecule is greater than the hydrogel pores. In erosion-controlled hydrogels, the release is governed by bulk or surface erosion.

### 4.9 Injectability

Injectable hydrogels, in particular, have the potential to improve non-invasive localised administration of medications, reliable and tailored placement, and specific administration to difficult-to-access tissue sites and interface tissues. The sol-gel state of transformation refers to the critical point at which a polymer solution transforms from liquid to solid. Injectable hydrogels, particularly *in situ* developing and shear-thinning hydrogels, experience a quick sol-gel phase shift, allowing the matrix to easily take on the form of the cavity, giving an ideal alignment and interface in tissues (Rizzo and Kehr, 2021). The main benefit of injectable scaffolds over pre-formed scaffolds is injectability, meaning that they can be injected with a syringe into the defect area and then hardened in place. They can fluidize under shear stress and then restore their mechanical characteristics, making them promising for uses in tissue regeneration. They can momentarily fluidize under shear stress before returning to their initial architecture and mechanical characteristics once the force is released. These injectable hydrogels can be utilized for locally targeted and prolonged pharmaceutical delivery, and their shear-thinning plug flow in syringes promotes the injection of live cells (Gaffey et al., 2015; Buwalda et al., 2017). Nevertheless, there is a growing desire for biomaterials that can briefly fluidize, i.e., have a shear-induced viscosity drop of the order of many years, accompanied by self-healing and restitution of their inherent mechanical characteristics (Guvendiren et al., 2012; Uman et al., 2020). The desire to distribute biomaterials in a non-invasive approach has fueled this research. When performing minimally invasive procedures, the injectability of a biomaterial is critical. Optimally, the material should be flowable before injection and quickly become immobile when it disperses throughout the deformity. The shift from solution to gel in hydrogels can satisfy that need. Temperature, pH, light, enzyme, or including a crosslinker can all trigger this transformation (Tahmasebi et al., 2023).

The injectability of a hydrogel is often connected to the rheological characteristics of the monomers or pre-polymers, and parameters including quantity, viscosity, gelation mechanism, and gelation pace all impact it. A pre-gelation solution’s concentration and viscosity should be suitable to minimize premature gelation while maintaining ease of operation (Hou, De Bank, et al., 2004). To retain maximum cell survival and molecular bioactivity while avoiding harm to adjacent tissues, a light gelation technique is preferred. A proper gelation rate is also critical; a quick pace may inhibit the spreading of the hydrogel and surgical operations, whilst a slower pace could compromise the hydrogel’s viability.

### 4.10 Adhesion

Mucoadhesion is typically defined as the adherence of two components, at least one of which has a mucosal surface. Over the last few decades, mucosal medication delivery has gotten a lot of interest. Mucoadhesive formulations may be engineered to allow for extended uptake at the location of administration, resulting in a regulated pace of drug dispersion and an enhanced therapeutic effect. This may be advantageous for pharmacological molecules that are not suitable to oral administration, including those that undergo acid degradation or substantial first-pass metabolism. The mucoadhesive capacity of a form of injection is determined by a number of parameters, notably the kind of mucosal tissue and the physicochemical qualities of the polymeric formulations (Vigani et al., 2023). Polymer adherence is a key feature that renders them more compatible with biological beings. It is determined by the inner composition and chemical interaction of the materials, whereas hydrogels have a considerable amount in the center, making them inferior when it comes to adhesiveness owing to hydrogen bonding (Bovone et al., 2021). Extensive retention of water in the hydrogel’s nucleus results in poor interaction with the substrate, leading to a space between the hydrogel’s actual surface and the substrate (Mortier et al., 2022). Additionally, water molecules establish hydrogen bonds with the hydrogels’ sticky regions, limiting the hydrogels’ interfacial contact with the desired substrate. Since most tissues are damp and delicate, using hydrogels in biomedical research exacerbates the issue (Yao et al., 2022). Bioadhesive injectable hydrogels have sparked a lot of significance lately owing to their extraordinary features. The various uses of these hydrogels, which range from wound recovery and reconstruction of tissues to attachment of cells and wearable sensors, are explored, highlighting their prospective significance in biomedicine and providing useful insights for future study (Xiong et al., 2021). Several research highlight the growing importance of bioadhesive injectable hydrogels in medicine. Investigators tried several approaches to solve these difficulties and took the intended texture into account before manufacturing the hydrogels (Bal-Ozturk et al., 2021). Since surface texture interaction with cells and tissues is required in practically all uses in medicine. Since thermo-polymers are intrinsically sticky, thermosensitive hydrogels displayed superior adhesion characteristics over regular hydrogels. So, as opposed to other standard materials, temperature-sensitive hydrogel might be regarded as a promising contender to address these stickiness concerns within conventional hydrogels (Klouda and Mikos, 2008).

## 5 Biomaterials used to construct injectable thermosensitive hydrogels

### 5.1 Natural polymers

Natural polymers formed from natural sources, such as polysaccharides and proteins, are biocompatible and biodegradable, rendering them an excellent alternative for the production of hydrogels. Polysaccharides utilized in hydrogel production include cellulose, chitosan, dextran, alginate, hyaluronic acid, and its derivatives. Polysaccharide chains contain several hydroxyl groups and/or other functional groups (amino, carboxyl groups, and so on), providing diverse potential for the creation of polymer-based hydrogels *via* chemical or physical cross-linking. Proteins include collagen, gelatin, and fibrin are essentially polymers of amino acids. It is believed that proteins may establish scaffolds through intermolecular and/or intramolecular stresses (including hydrogen bonds, electrostatic forces, and hydrophobic impacts) as well as constitute three-dimensional hydrogels *via* self-organized processes and wrapping under suitable circumstances (Ahn et al., 2021). Hydrogels made of natural polymers come from renewable sources. Natural polymers are remarkably biocompatible and biodegradable. Their hydrophilicity facilitates cell adhesion, proliferation, and differentiation. Natural polymers do not have the same mechanical durability and resilience as artificial hydrogels, which could restrict their applicability (de Santana and de Santana, 2015; Holiel et al., 2021).

#### 5.1.1 Polysaccharides

Polysaccharides are the most prevalent naturally generated macromolecular polymers derived from organic origins including algae, plants, and microbial chosen strain cultures. They are frequently employed as medicinal product carriers and tissue scaffolds because of their exceptional biocompatibility and biodegradability (producing body-favorable metabolites during disintegration). Furthermore, they have significant benefits regarding a similarity to biological macromolecules that can be identified by cellular ligands and improve cell adherence, propagation, and multiplication in regenerative applications (Atia et al., 2023b).

##### 5.1.1.1 Chitosan

Among them, natural polymers such as chitosan have been extensively used to construct several types of mucoadhesive drug delivery systems with successful treatments. Chitosan is a naturally existing biopolysaccharide that has been utilized in mucoadhesive devices owing to its strong adhesion properties. Chitosan’s mucoadhesivity could be described by the electrostatic contact between its positively charged cationic molecules and the negatively charged mucin (Hasnain and Nayak, 2022). However, chitosan’s insufficient aqueous solubility at both basic and neutral pHs is one of the most significant limitations to its application as a biopolymer in mucoadhesive networks. To address this issue, various chemical alterations to chitosan have been researched and effectively implemented (Atia et al., 2022a). CS’s inherent characteristics (molecular mass, extent of deacetylation) determine how it will be used. Peng et al. employed N-acetylation to create an acetylated carboxymethyl CS for inclusion into an intelligent hydrogel for periodontal therapy. His research revealed that by varying the level of acetylation of the carboxymethyl CS hydrogels, the gelation temperature could be adjusted (Peng et al., 2023). The gel formation temperature was 36.9°C when the extent of acetylation was 63.9%, and 33.2°C when the percentage of acetylation was 85.3%.

It has just been discovered that introducing hydroxyl-containing polymers including β-GP may end up in thermo-sensitive CS-based hydrogels. The hydrogel formed by mixing CS and -GP is frequently employed in the medical area, and this approach has been extensively utilized after Chenite originally described it. (Chenite et al., 2000). Since the hydrophobic and hydrogen linking forces surpass the electrostatic repulsive power, certain portions between the CS chains develop physical bonding when the hydrogel reaches gelation temperature (Molinaro et al., 2002).

Shen et al. encapsulated dental pulp stem cell-derived exosomes in a CS and -GP crosslinked hydrogel to treat periodontitis (DPSC-Exos/CS) (Shen et al., 2020). This hydrogel solidified at 37°C, and the bulk of the packed exosomes were ejected from DPSC-Exos/CS within 7 days in PBS at 37°C, with an exosome loading efficiency of around 80%. RNA sequencing investigation of periodontitis model mice subjected to DPSC-Exos/CS and CS indicated that genes linked with chemotactic processes, inflammatory reactions, and immunological reactions were decreased in periodontitis mice treated with DPSC-Exo/CS.

In comparison to the traditional gelation approach, the introduction of gelatin (Gel) enables the cross-linking of CS and β-GP via cation-anion electrostatic forces, reducing gelation time (Tóth et al., 2021). Xu et al. developed an injectable thermo-sensitive hydrogel containing CS, β-GP, and Gel loaded with aspirin, and erythropoietin for periodontal regeneration by regulating the inflammation-producing milieu and stimulating tissue regeneration. This hydrogel demonstrated prolonged aspirin and erythropoietin releasing (Xu et al., 2019). *In vitro* release tests verified these features, with aspirin and erythropoietin release rates of 86.6% and 69.4%, respectively, during the first 3 days. It maintained aspirin discharge at its initial stage, creating a regional milieu free of inflammation and boosting erythropoietin’s pharmacological action.

Zhang et al. incorporated HA into a CS/β-glycerophosphate sodium system (Zhang et al., 2018). The addition of HA alters the structural characteristics of the hydrogel and could minimize pore dimensions, which could be used to modulate the pharmaceutical distribution speed. Because HA molecules possess a substantial amount of hydroxyl, carboxyl, and amino groups, they may engage more strongly with CS, increasing its durability.

The architectural impact of β-GP during warming enhances hydrophobic contacts and connection between the CS and HA chains, and the solution transforms into a hydrogel because of the synergistic impact of these ionic reactions. Furthermore, the inclusion of HA has been shown to enhance the hydrogel formed *in situ*’s adhesion (Deng et al., 2017).

##### 5.1.1.2 Curdlan

Curdlan is a polysaccharide derived from microbes that comprises 300–500 glucose units connected by −1,3-glycosidic bonds (Zhang and Edgar, 2014). Without the addition of crosslinking substances, curdlan may undergo physical gelation by warming to generate a high-set thermally irreversible gel (80°C) and low-set thermally bidirectional gel (55°C). Curdlan, as a result, holds enormous potential for application in periodontal regeneration. Tong et al. employed photothermal treatment to obtain regulated and proper discharge of antimicrobial medicines following near-infrared (NIR) light stimulation (Tong et al., 2020). They mixed the antibacterial agent chlorhexidine acetate and the photothermal agent polydopamine (PDA) with curdlan. After 50 s of radiation, the hydrogel’s temperature increased to about 58°C, allowing it to eradicate 74.4% of the total bacteria (*Staphylococcus aureus*) in three light cycles with just minor tissue damage.

Compound hydrogels have been shown to have stronger retaining capabilities, a smaller swelling volume, and improved stability. Human periodontal ligament cells are also biocompatible with compound hydrogel. Furthermore, the photothermal agent’s cumulative and locally induced release improves the bactericidal action. This combinatorial antibacterial technique expands curdlan’s capabilities, enabling it to effectively combat periodontitis with more complicated treatments that concentrate and react to outside factors or its regional environment.

##### 5.1.1.3 Cellulose

Cellulose is the most common carbohydrate found in nature (Seddiqi et al., 2021). It garnered substantial interest as a desirable replacement material, because of its beneficial features, including being harmless, biodegradable and biocompatible, chemical durability, low prices, and significant hydrophilic nature, which makes it an ideal solution for the production of biologically compatible hydrogels (Ciolacu and Suflet, 2018). Venkatesh et al. used Pluronic F-127 and hydroxyethyl cellulose as the hydrogel matrix to create a thermo-responsive hydrogel preloaded with azithromycin to facilitate the cure of periodontitis (Venkatesh et al., 2013). The polyoxyvinyl chains of Pluronic F-127 interact with hydroxyethyl cellulose to promote dehydration, improve tangling with surrounding molecules, and significantly increase hydrogen bonds between molecules. As a result, it may be possible to lower the gelation temperature and use a hydrogel to treat periodontitis more effectively. Rheological investigations indicate that this hydrogel’s density increases with temperature, reaching a notable peak at 37°C.

This hydrogel also features a quick gelation time and an excellent gelation temperature. This hydrogel’s gelation temperature is lower than body temperature (29.8°C–33.1°C), and the time necessary for the hydrogel to attain the gelation temperature is between 33 and 42 s.

##### 5.1.1.4 Xyloglucan

Xyloglucan is a carbohydrate present in higher plant cell walls along with numerous seeds. It is of the highest quality biocompatible material that is readily available and biodegradable. When contrasted to other hydrogels, it has been extensively explored because of its higher capability for taking in and keeping water, as well as its superior biocompatibility. It has piqued research curiosity in the creation of hydrogels for biological purposes (Kulkarni et al., 2017). It was also lately tested as a potential cell transport vehicle via the addition of poly-D-lysine, which under physiological conditions caused a sol-gel transformation (Simi and Abraham, 2010). Cell confinement in regenerative medicine purposes requires the existence of microporous, three-dimensional structures that have connections (Silva et al., 2013; Ajovalasit et al., 2018).

##### 5.1.1.5 Agarose

Agarose is a seaweed-derived carbohydrate (Chudasama et al., 2021). This polysaccharide’s molecular arrangement is left-handed triple-double helices, which allows it to produce highly durable gels even at small amounts (Utech and Boccaccini, 2016). Agarose has a fascinating thermosensitivity: depending on the type of agarose, its polymer chains can display a random coil structure at high temperatures (N65C-95C), and they can also form double helical aggregates at around 35°C (Zeng et al., 2015). It is water soluble at different temperatures and is extensively used as a cell immobilization matrix, pharmaceutical carrier, and cellular incorporation. These hydrogels’ interconnecting porosity morphologies, excellent mechanical strength with moderate biodegradation, extraordinary tissue adhesiveness, and self-healing potential render them attractive options for long-lasting wound dressings (Zeng et al., 2015).

##### 5.1.1.6 Hyaluronic acid

Hyaluronic acid (HA) is an organic polymer. The physiological effects of HA can be divided into two categories: signaling molecules and passive architectural molecules. As an extracellular substance, HA stabilizes the space by forming a hydrated and organized system while also changing tissue hydration and osmotic equilibrium. It can, on the other hand, function as a signaling molecule, interacting with binding proteins. By being highly biocompatible, biodegradable, and mechanically viscoelastic, HA-based hydrogel not only retains the biological capabilities of HA but also endows the hydrogel with skeletal functioning. In order to boost the efficacy of BMP-2 in bone therapy, injectable substances that are safe and break down in a controlled way, including HA, with increased vehicle qualities were created (Patterson et al., 2010). HA was chosen as a sample of mucoadhesive compounds. This choice is significant because, in some ways, HA represents the worst-case scenario for mucoadhesive polymers. In fact, HA has been regarded a borderline material that may have particular reactions with cellular elements when delivered through routes other than mucosal regions (Vasvani et al., 2020).

##### 5.1.1.7 Starch

Starch is a plant-derived material that is frequently engaged in bone tissue engineering and medicine delivery. Swelling capabilities, rheological features, enzymatic digestion, shape, and solubility are some of its major characteristics that increase its application in drug delivery studies. As a result of differing manufacturing processes, starch may associate into an array of semi-crystalline forms and construct numerous hydrogel formulations. Native starch granules absorb water and expand when heated in excess water. Following the permanent loss of granule structural and crystalline order, amylose leaches into the surrounding media, a method known as starch gelatinization (Nikolenko et al., 2023).

Starch has considerable potential for the creation of “green” hydrogel-based products since it is an accessible, sustainable, and ecologically friendly substance with a diverse spectrum of rheological, chemical, physical, and biochemical characteristics (Bertoft, 2017). For hemostatic usage, a new Kappa carrageenan (CA)-coated Starch/cellulose nanofiber (CNF) with customizable biomechanical characteristics. The durability of CA-coated Starch/CNF hydrogels was greatly increased (up to 2 times) when compared to Starch/CNF hydrogels, depending on the CA concentration.

Because of the collaborative impacts. of starch/CNF hydrogel and CA coating, remarkable features like improved mechanical capabilities, tunable rates of decomposition, and blood coagulation capacity were obtained, making CA-coated starch/CNF hydrogel a suitable choice for hemostasis (Tavakoli et al., 2021). Starch/Chitosan Gelation duration and compressive modulus studies revealed that the particles had a substantial impact on the mix network density and hydrogel mechanical characteristics (Baniani et al., 2017). The freeze-thaw process was used to create antibacterial starch-citrate hydrogels. This hydrogel showed remarkable antibacterial action against Gram-negative bacteria strains such as *E. coli, Staphylococcus pyogenes, Salmonella thypimurium,* and *S. aureus*. As a result, the potential utilization as both an antibacterial agent and a drug delivery carrier is predicted (Chin et al., 2019). Cardoso et al. (de Oliveira Cardoso et al., 2017) created hydrogels from gellan gum and starch retrograded combinations for pharmaceutical delivery purposes by adjusting polymer and cross-linker proportions. Structural changes to the retrograded starch mix hydrogels were achieved through isothermal cycles at 4°C or alternate thermal cycles. Under chilling temperatures (4°C/8 days), hydrogels developed a more organized and resilient architectural system, resulting in a tougher and more cohesive substance. Under cycled temperatures (4°C and 30°C/16 days), hydrogels formed a looser network with more mobility and increased adhesiveness. Furthermore, Cardoso et al. (de Oliveira Cardoso, Cury, Evangelista and Gremião, 2017) showed that these hydrogels may be tailored to have appropriate adhesiveness, durability, and flexibility, making them suitable compounds for mucoadhesive devices for drug delivery. Dong et al. (Dong et al., 2019) also created a starch-based thermosensitive hydrogel that exhibits reversible swelling and deswelling as the temperature varies. They found that combining hydroxybutyl starch and PEG resulted in more porous architectures and enhanced the swelling ratio. Furthermore, these hydrogels displayed recurrent swelling at 25°C and deswelling at 45°C.

##### 5.1.1.8 Alginate

Alginate (AG) is an organically existing anionic polymer composed of 1,4-linked-D-mannuronic acid and -L-guluronic acid that generally comes from brown seaweed. It has been deeply studied and utilized in numerous fields of medicine due to its biocompatibility, low toxicological profile, affordable price, and straightforward gelation ability *via* the introduction of divalent cations like calcium (Ca^2+^) (Chan and Neufeld, 2010; Alkhursani et al., 2023). ALG is classed as an anionic mucoadhesive polymer because it has carboxyl groups that may interact with the hydroxyl groups of mucin glycoproteins. Furthermore, ALG has gelling characteristics. Despite the numerous benefits of ALG, ALG-based drug delivery devices have disadvantages such as poor flexibility, inadequate mechanical characteristics, and brittleness. As a result, novel strategies to improve the properties of ALG preparations are required (Szekalska et al., 2016; Kruk and Winnicka, 2022). COL and AG can operate together to bring together their beneficial attributes and overcome the limitations associated with every substance.

The key barriers limiting their wide range of applicability are the poor mechanical characteristics of COL and the intrinsic absence of cell-binding motifs within AG, which may be solved by combining them with increased cell-binding motifs and boosted durability. Furthermore, the simplicity with which these composite gels under moderate circumstances allows for the preservation of bioactive substances and improves cell encapsulation (Hu and Lo, 2021). Nerve growth factors (NGF) are significant in bone healing (Yada et al., 1994). Human NGF’s quick removal from the body via enzymatic breakdown is one of its limitations. To address this issue, NGF was added into a COL/nHAp/AG hydrogel and tested for bone formation potential in a rabbit mandibular distraction osteogenesis model (0.75 mm/12 h for 6 days). The results demonstrated the hydrogel system’s osteoconductive ability and that human NGF might maintain its biological functions for an extended amount of time after being released from the NPs/hydrogel combination (Cao et al., 2012).

##### 5.1.1.9 Gellan gum

Given its biological compatibility and breakdown, gellan gum (GG), a polysaccharide substance, is utilized to make injectable hydrogels (Ahmed, 2015). GG is a thermally responsive biomaterial with a UCST (ultimate critical solution temperature). The GG solution turns into the gel at temperatures lower than this. Additionally, the elevated carboxyl groups in GG particles have the potential to react with ambient calcium ions and trap local Ca ion levels, which is advantageous for bone regeneration. Injectable thermosensitive hydrogel based on gellan gum and cross-linked with 2-methacrylamidoethyl dihydrogen phosphate (MDP) enhanced the survival of the preosteoblast MC3T3-E1 cell line and elevated the amount and rate of hydroxyapatite production (Li A et al., 2020).

#### 5.1.2 Natural proteins

##### 5.1.2.1 Albumin

Albumin is a well-known macro-molecule carrier that may be found in numerous foods. It facilitates in the delivery of nutrients to cells, in addition to the regulation of osmotic pressure (Loureiro et al., 2016). Nontoxicity, biodegradability, ease of manufacturing, no immunogenicity, with clearly established dimensions make albumin carriers widely implemented in tissue engineering Nontoxicity, biodegradability, ease of manufacturing, non-immunogenicity, with clearly established dimensions make albumin carriers are widely implemented in tissue engineering (Belinskaia et al., 2021).

##### 5.1.2.2 Gliadin

Gliadin is a Gluten protein obtained from wheat. Gliadin’s capacity to adhere to the mucus barrier makes it a popular ingredient in mucoadhesive treatments. Its exceptional features, which include being biodegradable material, its organic prevalence, safety, and reliability, render it an ideal choice for drug delivery systems. They can safeguard the loaded medicine for controlled and prolonged release due to their hydrophobic nature and solubility (Voci et al., 2021).

##### 5.1.2.3 Zein

Zein is a prolamine-rich protein that includes hydrophobic amino acids, proline, and glutamine. Zein has been authorized by the FDA for human consumption. Several medications and bioactive compounds have been encased in zein protein nanoparticles (Corradini et al., 2014).

##### 5.1.2.4 Fibrin/fibrinogen

Fibrin, a biodegradable and biocompatible protein, is generated by the polymerization of the enzyme fibrinogen. Fibrin is an excellent bone-healing agent (Riedelová-Reicheltová et al., 2016). Fibrinogen is a glycoprotein present in the blood that is composed of three pairs of non-identical polypeptide chains. After arterial damage, thrombin cleaves fibrinogen to create fibrin, the most prevalent constituent of blood clots. When blood coagulates because of an injury, fibrin gel can develop in a matter of seconds. Fibrinogen may react with cells via integrin receptors, which include two RGD sequences as well as non-RGD sequence integrin binding sites and non-integrin receptors (Joo et al., 2015), and this provides the fibrinogen hydrogel with great bio compatibility.

At biological conditions, fibrin gel forms easily and is suited for local administration via direct injection to the target location (Lei et al., 2009). Because fibrin may be derived from a patient’s plasma, immunological rejection is avoided. The fibrinogen cleavage byproducts and fibrin degradation substances are non-toxic and have diverse regulating impacts on cell division and development. They specifically increase angiogenetic and vasoactive activity, as well as the accumulation of collagen, during tissue healing (Herrick et al., 1999). Because of these benefits, fibrin is often utilized as a platform in tissue engineering.

Fibrinogen can operate as a repository for growth mediators, proteases, and protease suppressors by attachment to other ECM components. This interaction not only improves enzymatic attachment to the framework and cellular-mediated fibrinogen cleavage, but it also allows for the release of bioactive compounds in the immediate microenvironment (Karamanos et al., 2018). As a result, fibrinogen hydrogel may be employed as a bioactive carrier.

Fibrinogen hydrogel has limitations as a biological biomaterial, including weak mechanical properties and less predictable degradation. Copolymerization with artificial biomaterials allows for a certain degree of control over the fibrinogen hydrogel’s architectural features and biodegradation while preserving its intrinsic biocompatibility (Almany and Seliktar, 2005).

##### 5.1.2.5 Sericin

Sericin, a water-soluble and hydrophilic protein acts as a glue and accounts for 20%–30% of the cocoon’s mass. With the inclusion of sulphate, it has intrinsic capabilities such as anti-cancer, antioxidant, and coagulation inhibition as a sericin nanoparticle (Nayak and Kundu, 2016). Sericin is also non-toxic to fibroblast cells. The existence of cysteine and methionine amino acids promotes cell development (Cao and Zhang, 2016). Water-soluble silk sericin has little immunogenicity. Sericin demonstrates easy wound healing without the inflammatory process (Cao and Zhang, 2016).

##### 5.1.2.6 Keratin

Keratin, a structural protein that’s high in the amino acid cysteine, proclaims the greatest mechanical capacity. Keratin nanosuspension is employed as a coating to examine cellular multiplication, and it is a less expensive alternative to fibronectin or collagen. Furthermore, keratin hydrogels are employed in tissue engineering (Chen et al., 2022).

##### 5.1.2.7 Soy protein

The most plentiful plant-based protein resource is soybean (glycine max). Its isolates are an enhanced type of soy protein that has been shown to have excellent nutritional values and ingredient functions. Glycinin and conglycinin are significant components of soy protein isolate (Qin et al., 2022). Soy protein isolate aggregates when crosslinking chemicals are added, and at specific temperatures, microspheres, hydrogels, and polymer mixes develop.

##### 5.1.2.8 Casein

Casein is plentiful in milk and dairy products and is considered. Its nature enhances particle development, and crosslinking may be used to boost the resilience of their frameworks and their practical usefulness (Liu S et al., 2018).

##### 5.1.2.9 Silk fibroin

Silk Fibroin (SF) is an easy material to deal with, and its outstanding mechanical properties and controlled biodegradation render it an outstanding option for bone regeneration scaffolds (Saleem et al., 2020). The SF is composed of two components known as sericin and fibroin, and fibroin was employed to reduce inflammatory and allergic reactions following surgical procedures (Sun et al., 2021). SF scaffolds were previously adjusted with a range of osteogenic bioactive sources such as natural-derived materials, bone growth agents, and chemicals (Celikkin et al., 2017).

##### 5.1.2.10 Elastin

Elastin is a protein present in connective tissues that offers elasticity and toughness to tissues that need to be extensible and rebound. It is formed of hydrophobic domains containing residuals of glycine, valine, and proline, in addition to crosslinking domains including lysine and alanine (Wang et al., 2021). Natural elastin received fewer considerations for biomaterial applications than collagen due to the challenges in isolating it and the high chance of calcification upon transplantation (MacEwan and Chilkoti, 2010).

A temperature rise, for example, leads soluble protein to separate from the solvent and form an aggregated phase, whereas a fall in temperature increases the possibility of the protein self-aggregating (Costa et al., 2009). Elastin-like proteins (ELPs), which may be useful in tissue engineering and cancer therapeutics, have been recombinantly generated as oligomeric repeats of the pentapeptide sequence VPGXG, where X indicates an amino acid other than proline (Despanie et al., 2016). They are soluble below 35°C and form a gel-like matrix above body temperature (37°C) after injection. Consequently, ELP enables the integration of cells and other therapeutic agents into its solution state at room temperature, as well as the formation of a gel-like matrix at normal temperatures that can contain them for tissue engineering, cell therapy, and therapeutic purposes (Varanko et al., 2020).

##### 5.1.2.11 Collagen

In the ECM and mammals, collagen is the most abundant protein. Collagen may be derived from a wide range of mammals and tissues, therefore being extensively accessible for research and treatment. To minimize batch-to-batch variances and possible sensitization of animal-originated collagen, recombinant collagen has been examined as a replacement to resemble human collagen (Rezvani Ghomi et al., 2021). Collagen gels were initially employed as a means of skin regeneration before being extensively utilized for other regenerative purposes (Zhang et al., 2023). Furthermore, injectable collagen gels have been demonstrated to stimulate stem cell adipogenesis and osteogenesis (Hao et al., 2008). Collagen-based hydrogels have been created by either letting collagen fibrils self-assemble or employing chemical crosslinking reagents.

Rebuilding of the periodontal attachment apparatus was shown to be facilitated by implanting a three-dimensional scaffold composed of collagen hydrogel and shaped like a sponge, as shown by studies by Kosen et al. (Kosen et al., 2012), and Kato et al. (Kato et al., 2015). Adverse periodontal reactions, including as ankylosis and root resorption, were consistently absent when collagen hydrogel was used. Conversely, the amount of periodontal tissue that was growing was about half that of the experimental deficit. To induce periodontal rebuilding, an integrated strategy for tissue engineering is required.

##### 5.1.2.12 Gelatin

Gelatin is a possible naturally formed polymer for the development of hydrogels due to its non-antigenicity, nontoxicity, biodegradability, biocompatibility, and low cost (Amadori et al., 2015). Despite this, gelatin loses physical durability and flexibility *in vivo* due to continual water imbibition (Wisotzki et al., 2014). Consequently, unexpected waves of hydrogels occur. Additionally, gelatin’s heat stability is restricted, with the sol-gel transition happening at body temperature (37°C), constraining its practical application (Biswal et al., 2016). These problems can be solved by using strengthening agents and cross-linking procedures (Skopinska-Wisniewska et al., 2021). Attempts in the past to cross-link gelatin with glutaraldehyde have led to toxicological challenges. Genipin, a natural crosslinking agent, can be used as an ecologically benign alternative to glutaraldehyde; however, due to its high cost, its applicability is (Serpico et al., 2023).

Radiation-induced cross-linking, on the other hand, is a relatively simple and low-cost strategy that employs non-chemical substances and permits concurrent sterilization of the crosslinked polymer (Hafeez et al., 2018; Zainal et al., 2021). Gelatin’s chemical backbone produced a three-dimensional architecture by producing gamma rays via cross-linking (Wan Ishak et al., 2018). A multifunctional hydrogel constructed from homologous injectable platelet-rich fibrin (iPRF) and Gel NPs was established to boost its durability and delay decomposition while slowly discharging iPRF-entrapped growth factors (Mu et al., 2020). This organic copolymer has a DN framework, which increases mechanical properties including injectability, durability levels, and number of deformations. Sinus chambers injected with Gel NPs-iPRF hydrogels revealed considerably greater fresh bone development following four and 8 weeks of application in a sinus augmentation paradigm, histologically and radiologically than Gel NP gels and the untreated control, with a greater extent of angiogenesis. After 8 weeks, the newly produced bone developed and remodeled into lamellar bones (Mu, Chen, Yuan, Li, Huang, Wang, Zhang, Liu, Luo and Liang, 2020). As a result, it serves as a carrier for the prolonged liberation of bioactive ingredients from iPRF.

### 5.2 Composites

Composite biomaterials frequently exhibit an outstanding mix of strength and toughness, as well as increased properties when compared to their separate components (Solangi et al., 2023). The biomechanical responsiveness of the framework is affected by parameters like as its composition and ratio, porosity, and the addition of bioactive substances (El Sayed, 2023).

## 6 Pathophysiology of periodontitis

**FIGURE 4:**
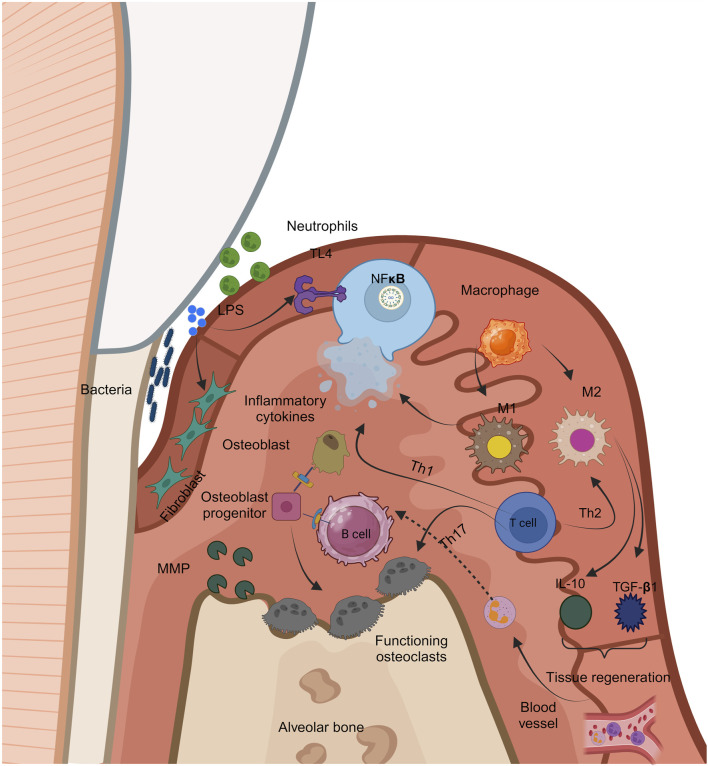
Figure 4 Immunological responses to periodontal inflammation.

Periodontitis, as previously stated, is a multifaceted condition that results from subgingival colonization of pathogenic microorganisms (Figure 4). The condition is caused by an imbalance between pathogenic bacteria colonizing the subgingival area and the effectiveness of the host’s regional and systemic defenses (Könönen et al., 2019a). Variations in the periodontium are influenced by the host’s contact with the bacterial flora, which can result in a variety of clinical manifestations of periodontitis. Periodontitis is distinguished by the development of periodontal defects. They are caused by inflammatory and dystrophic mechanisms that degrade connective tissue. Consequently, the gingival tissues withdraw apically, and the teeth stay exposed, giving the impression that they are longer. Furthermore, regarding dysbiosis, the existence of local and generalized variables is vital for disease progression (Hajishengallis, 2015).

Local variables include anatomical defects, dietary deficiency, inadequate sanitation, iatrogenic variables, and dental cavities, whereas general factors include blood illnesses, endocrine disorders, genetic factors, and aging. Furthermore, several risk factors, like smoking, diabetes, stress, drugs, food, hormonal variables, immunodeficient status, connective tissue disorders, and others, enhance the possibility of the onset and progression of periodontitis (Knight et al., 2016). The bacterial enzyme endotoxin allows germs to directly penetrate the gingiva, causing the pathogenic impact of the oral microbiome. Using enzymes, periodontal bacteria target intercellular spaces, followed by cells in the epithelium and connective tissue. It is believed that bacterial endotoxins are mostly to blame for the onset and development of periodontitis. Endotoxin induces proinflammatory chemicals, including prostaglandin E (PgE2), tumor necrosis factor-alpha (TNF-α), and interleukins (IL-1, IL-6, IL-8, and others), which compromise the immune system (Di Stefano et al., 2022).

Periodontitis often starts on the gingiva and progresses to the remainder of the periodontium. Gingival inflammation is a distinct condition that can extend throughout the remainder of the periodontium. There are four clinical phases: preliminary, early, developed, and advanced. In the advanced stage, the gingival lesion transforms into an alveolar bone lesion, resulting in the start of periodontitis (Martínez-García and Hernández-Lemus, 2021).

Interdental damages below the contact site cause the first lesion. An early lesion is associated with a rise in the quantity of pathogenic bacteria and a rise in the degree of inflammatory infiltration, that physiologically contributes to gingival expansion. At this point, the devastation begins. The formed lesion appears 2-3 w following the start of the buildup of the dental biofilm, at which point the clinical characteristic of moderate or severe inflammation begins. The loss of collagen is characterized by an upsurge in the number of cells. Owing to the higher quantity of collagenase in irritated gingival tissue, collagen degradation is higher. There is a lack of connective tissue connection along the root surface at the advanced lesion stage, as well as substantial apical migration of the junctional epithelium and the emergence of ulcerations (Visentin et al., 2023).

Periodontal pocket development happens clinically, and chronic periodontitis occurs. The emergence of periodontal pockets is the pathological and essential indication of chronic periodontitis. These are pathological structures generated by tissue degradation that deepen the gingival sulcus, and they only develop in people with periodontitis. Periodontitis can lead to tooth deterioration and tooth loss. It is feasible to manage periodontal disease by understanding its etiology and the clinical changes that follow it (Bosshardt, 2018).

## 7 Currently implemented periodontal therapeutic modalities

Periodontal treatment is a lengthy and complex procedure. Its purpose is to avoid, manage, and eliminate periodontal diseases. The goal of contemporary periodontal therapy is to have total periodontium regeneration. Periodontal treatment is tailored to everyone. The treatment plan should contain all therapeutic methods that will aid in achieving the best morphological, and biological conditions in the mouth. The treatment approach must involve patient encouragement and compliance, in addition to recommendations on proper dental hygiene. It is also necessary to do preliminary treatment to eradicate acute or uncomfortable problems. Following the preparatory phase, the treatment is administered in the prescribed order. It is divided into four stages, beginning with causative therapy, moving on to surgery, and concluding with occlusal balance and surveillance of patients (Ivanovski, 2009).

### 7.1 Phase-I therapy

The initial step of conventional treatment is causal therapy. It entails the elimination of dysbiosis as well as the variables that speed up the formation and activity of the microbiome. The second component of the traditional treatment targets periodontal pockets using mechanical curettage of the pocket’s soft and hard walls. In addition to causative therapy, symptomatic therapy, which involves the removal of illness symptoms, is utilized. The current strategy also includes addressing risk factors (Kuraji et al., 2023).

### 7.2 Surgery

As the second stage of therapy, surgical therapy enables greater accessibility to the addressed soft, and hard tissues. The objective of surgery is to eradicate the origin of the illness, decrease periodontal pockets, and allow for optimum anatomical and functional tissue restoration. Surgical treatment is classified into several forms (Graziani et al., 2018).

#### 7.2.1 Regenerative surgery

The goal of regenerative surgery is to reestablish damaged periodontium by promoting attachment. In clinical dentistry, the concepts of periodontal tissue regeneration depend on passive and active regeneration approaches. The most frequently employed techniques for the inert regeneration of intraosseous periodontal defects involve the incorporation of biological molecules, in addition to the utilization of biological membranes in guided tissue regeneration (Ramseier et al., 2012). Additionally, participative periodontium regeneration involves tissue engineering, which integrates stem cells and growth factors in a suitable matrix, and biologic mimicry, which imitates normal periodontal development processes using biomolecules like enamel growth protein. Substances used in regenerative periodontal surgery must be biocompatible, corrosion-resistant, and easy to handle, and should minimize operational and postoperative issues while providing reliable therapeutic results.

The word “regeneration” applies to the repair of damaged or wounded tissues in a manner that favors the production of fresh attachment, such as the development of a new periodontal ligament with its fibers inserted in freshly produced tissues (Siaili et al., 2018). Regenerative therapies, unlike traditional anti-inflammation periodontal therapy, attempt to replace damaged periodontal tissues making it a harder mission. Several approaches have been investigated in conjunction with regenerative biomaterials, including hard- and soft-tissue grafts or cell-occlusive barrier membranes (Siaili et al., 2018).

##### 7.2.1.1 Implants and bone transplants

In order to preserve the surrounding tissue, preserve the shape of the defect, and reduce distortion of the adjacent tissue, the passive regeneration of periodontal tissues through the use of bone grafts and inserts is based on the architectural reinforcement of the affected area. Stem cells or progenitor cells with the potential to differentiate into osteoblasts are used in osteogenic bone grafts and implants. They could also be osteoinductive, meaning they supply substances that stimulate the differentiation of pluripotent stem cells, which in turn promotes the formation of new bone. In the most basic case, the implants might be osteoconductive, meaning that their chemical and biological properties promote the formation of new bone. Bone grafts and other bone replacements are categorized as autologous, allogeneic, xenogeneic, or alloplastic based on their biological makeup (Reynolds et al., 2010).

##### 7.2.1.2 Autogenic grafts

Bone grafts that are autogenous, or self-generated, are taken from the patient and are believed to possess ideal osteogenesis, osteoinduction, and osteoconduction qualities. The extraoral or intraoral donor sites are the sources from which they can be received. Schallhorn et al. were the first to use the iliac crest as an extraoral bone transplant in periodontal regeneration surgery in the late 1960s. However, iliac bone marrow transplantations are no longer used because of the invasive extraction procedure (Sakkas et al., 2017).

##### 7.2.1.3 Allogeneic grafts

Because allogeneic transplants are extracted from different humans, they offer some risks owing to the potential of antigenic properties (Sohn and Oh, 2019).

##### 7.2.1.4 Xenogeneic grafts

They are natural materials derived from animals that have had their organic parts eliminated to retain the bone framework in its unaltered inorganic state. Antigenicity is a persistent concern in allogeneic transplants, as it is in allogeneic grafts.

##### 7.2.1.5 Alloplastic implants

Alloplastic transplants are biocompatible artificial bone replacements with osteoconductive characteristics. These materials are classified as resorptive or non-resorptive based on their resorption (Eppley et al., 2005).

##### 7.2.1.6 Guided tissue regeneration

Guided tissue regeneration is the second stage of treatment for mucogingival and periodontal disease. Restitution ad integration, or the complete structural, morphologic, and histologic regeneration of the injured periodontal tissues, is the aim of the treatment. In the early 1980s, the idea of selectively controlling the regeneration of periodontal tissues by the use of a membrane positioned between the gingiva and the tooth surfaced (Uskoković et al., 2022). The notion of directed tissue regeneration suggests that the apical growth of the epithelium is stopped or prevented, allowing room for regeneration. Periodontal ligament cells can develop into fibroblasts, cementoblasts, or osteoblasts, allowing them to establish new attachments.

For directed tissue regeneration, three kinds of membranes are employed: natural, which degrades naturally, resorptive artificial, and non-resorbable artificial. An ideal membrane should be safe, that is, biocompatible and solid, incapable of transferring illness or collapsing into a defect, yet able to distribute antimicrobials, bioactive molecules, and other additional agents (Naung et al., 2019).

## 8 Treatment of periodontitis *via* employing injectable thermos-responsive hydrogels

The choice of the proper treatment material and method of delivery are critical in treating periodontitis. Regarding dose form, hydrogels are the best local delivery mechanism for periodontal locations.

### 8.1 Getting rid of pathogenic microbes

The deterioration of periodontal tissue begins with oral bacterial infection, and periodontitis develops as a consequence of an association between the microorganisms and the host (Bui et al., 2019). The greater part of plaques is made up of normal oral flora, with just a tiny percentage made up of highly aggressive pathogens causing direct or indirect tissue damage. Microbial antigens stimulate a variety of host defense cells, resulting in the production of several inflammatory substances and a regional immune reaction which causes secondary damage to periodontal tissues. With these plaque characteristics in consideration, current periodontal treatment focuses on balancing the microbiome and the harmful microbes, rendering antibacterial a valuable adjuvant in periodontal therapy (Bui, Almeida-da-Silva, Huynh, Trinh, Liu, Woodward, Asadi and Ojcius, 2019).

The draw backs of antibiotic therapy, on the other hand, are obvious: the incapability of a large amount of the drug to make its way more deeply into the periodontal pocket, a greater likelihood of side effects, a boost in infectious bacteria that develop resistance to drugs, and undetermined adherence among patients (Barca et al., 2015). According to existing studies, there are novel antimicrobial loaded injectable thermos-responsive hydrogels available for periodontitis, and are commonly used for these reasons since they have high antibacterial characteristics (Table 2). Popescu et al. (Popescu et al., 2022) used a twofold crosslinking technique including freeze-thaw cycles to create polyvinyl alcohol (PVA)/CS hydrogels incorporating silver nanoparticles. The most effective antimicrobial activity was identified towards *S. aureus*, and the blocking impact towards Pg enhanced as the proportion of silver nanoparticles in the hydrogel grew, suggesting that silver nanoparticle packed PVA/CS/OA might be utilized for managing periodontal disease.

**TABLE 2:** Application of injectable thermosensitive hydrogels to deliver antimicrobial agents.

Hydrogel	Main polymer	Bioactive agent	Model	Outcomes	References
Curdlan/polydopamie		Chlorhexidine		Bactericidal activity	Tong et al. (2020)
ZnO-NP/Chitosan/β-Glycerophosphate			*In vitro*	Temperature sensitivity and bacteriostatic	Huang et al. (2022)
CHX/β-CD/CS/BMP-2/alginate	β-CD/CS/	Chlorhexidine rhBMP-2		CHX and rhBMP-2 are released in a controlled manner	Zhou et al. (2022)
Chitosan/gelatin/-glycerophosphate	chitosan	Metronidazole, and vancomycin hydrochloride	*In vitro*	Thermosensitive at 37OC.	Pakzad and Ganji, (2016)
				Burst release of vancomycin hydrochloride, sustained release of metronidazole	
Chitosan/vanillin		ornidazole and doxycycline hyclate	Ligature-induced periodontal rat model	Antimicrobial activity is biocompatible and biodegradable	Yadav et al. (2018)
VH-HA-CS/BGP/TCP		Vancomycin TCP		Antibacterial characteristics	Li Y et al. (2020)
Chitosan/quaternized chitosan/α,β-glycerophosphate		Ornidazole		Antibacterial activity	Ji et al. (2009)
Chitosan	antimicrobial peptides (Nal-P-113) and/or antioxidants (polydopamine nanoparticles, PDNPs)			Sustained drug release for 13 days. Bactericidal activity	Ma et al. (2022)

Biogenic silver nanoparticles (Bio-AgNP), Zinc oxide nanoparticles (ZnO-NP), microspheres loaded *in situ* gel (MLIG), Vancomycin hydrochloride/Hydroxyapatite/chitosan/Beta glycerophosphate/Tricalcium phosphate (VH-HA-CS/BGP/TCP), Egg yolk immunoglobulin (IgY), human antimicrobial peptide LL-37, a modified antimicrobial peptide (Nal-P-113).

The next are some theories addressing how copper nanoparticles limit development in bacteria. For starters, the buildup, and the dissolution of copper nanoparticles in bacteria, result in the release of LPS, membrane proteins, and internal biological molecules, in addition to proton motive force generation at the plasma membrane. Nano copper is also ingested by cells, reducing internal ATP generation and halting replicating DNA (Wang et al., 2017). Gonz’alez et al. created an antibiotic release method for periodontal treatment using CS and copper nanoparticles and tested its antibacterial effectiveness against Aa *in vitro* (González and Cádiz, 2017). The copper nanoparticles/CS hydrogel showed a significant inhibitory halo against Aa.

Zinc (Zn) has been shown to impede microbiological activities (Pasquet et al., 2014). Electrospinning was used to create composite membranes of polycaprolactone, and Gel filled with ZnO nanoparticles. Antibacterial tests revealed that the existence of Zn nanoparticles considerably reduced the development of *S. aureus*. Human osteoblasts and HGF were also biocompatible with the Zn nanoparticles (Permyakova et al., 2022).

### 8.2 Periodontal inflammation environment regulation

Although infectious organisms are the origin of periodontitis, the inflammatory microenvironment has a significant impact on the disease’s progression. To remove bacteria, immunological cells (macrophages and T cells) in the periodontal milieu are activated and produce different cytokines (IL-1, IL-6, TNF-α) and ROS; Therefore, inflammation channels of communication are arbitrarily boosted and disturbed. This harms normal periodontium and encourages inflammation establishment, progression, and development (Han et al., 2023). That`s why injectable thermosensitive hydrogels loaded with anti-inflammatory agents, appear as promising candidates in non-surgical periodontal therapy (Table 3).

**TABLE 3:** Application of injectable thermosensitive hydrogels to deliver anti-inflammatory agents.

Hydrogel	Bioactive agent	Model	Outcomes	References
Thermally responsive chitosan	statins (atorvastatin and lovastatin) nano emulsions	*In vitro*, and *in vivo*	*In vitro*, cytocompatibility, biodegradable, reduced production of pro-inflammatory markers (TNF-α and IL- 1)	Petit et al. (2020)
			*In vivo*, increased neo bone formation	
Acetylated carboxymethyl chitosan	Caffeic acid phenethyl ester	Human periodontal ligament stem cells (hPDLSC)	Block TNF-α, IL-1, IL-6, and IL-17 production during inflammation	Peng et al. (2023)
			Boost osteoblastic differentiation in human periodontal ligament stem cells	
Sodium alginate/methylcellulose/poloxamer 407	Dried Scutellaria baicalensis extract/Cs		Intelligent hydrogels improve hyaluronidase resistance, metals removing, and antimicrobial action	Chanaj-Kaczmarek et al. (2021)
CS/β-GP hydrogel	Interleukin-1β (IL-1β) is a crucial pre-inflammatory factor	periodontal Inflammation in Diabetic Rats	Inhibited periodontal inflammation and reduce alveolar bone absorption	Liu et al. (2022)

Tumor necrosis factor (TNF-α), interleukin (IL), interleukin-1β (IL-1β), reactive oxygen species (ROS), doxycycline (DOX) and/or lipoxin A4 (LXA4), polyisocyanopeptide (PIC), chitosan (CS) Betaglycerophosphate (β-GP).

#### 8.2.1 Control of inflammation cells

Macrophages are the initial line of defense in the immune response and a critical type of cell during periodontitis (Yin et al., 2022). Meloxicam (Mex), a specific anti-cyclooxygenase 2, is a very effective nonsteroidal anti-inflammatory drug (NSAID). Mex, when injected subcutaneously, may reduce alveolar bone loss in rats with periodontitis (Bezerra et al., 2000).

Furthermore, Mex is a strong inhibitor of acute exudation in periodontal tissues as well as osseous degeneration. Chronic periodontitis can be greatly alleviated by mex-loaded temperature-sensitive gels (Rein et al., 2020). A month following therapy with Mex-loaded gels, the plaque index, gingival index, and probing depth all reduce dramatically (Rein et al., 2020). Ibuprofen/heparin/poly (N-isopropylacrylamide) injectable hydrogel was employed by Andrgie et al. to assist minimized discomfort and elevated inflammation during recovery. They employed the BALB/c mouse to assess its impact on inflammation resolution, and proinflammatory mediators production (Andrgie et al., 2020).

#### 8.2.2 Management of proinflammation mediator’s oxidative species

Cytokines are important controllers of immunological responses, and their abnormalities can cause or worsen periodontitis. Periodontitis-induced inflammatory variables can promote systemic inflammatory processes, affecting blood glucose regulation and the onset and progression of diabetic problems (Ramadan et al., 2020).

IL-1 is a cytokine with multiple functions that have been linked to the etiology and progression of periodontitis and diabetes. Throughout the course of periodontitis, fibroblasts are stimulated by IL-1 to release collagenase, interstitial lytic enzyme, and gelatin-degrading enzyme, which leads to matrix degradation, connective tissue loss, and periodontal tissue death. Moreover, IL-1 can stimulate the differentiation of periodontal ligament mesenchymal stem cells towards osteoclasts and induce osteoblasts to secrete prostaglandin E2 and plasmin, which increases inflammation and bone absorption (Cheng et al., 2020).

IL-1ra can reduce the biological functions of IL-1 by binding with the IL-1 receptor, lowering the generation of inflammatory molecules which include IL-6 and TNF-α and suppressing the formation of osteoclasts (Dinarello, 2011).

Thus, a temperature-sensitive CS, β-GP, and gelatin (gel) hydrogel containing IL-1ra was created, examined, and its ability to reduce inflammation in high-sugar settings was established (Liu et al., 2022). The hydrogel gelled at 37°C for 5 min, with pore sizes ranging from 5 to 70 m. On the 21st day, the total amount of IL-1ra released was 83.23%. Real-time polymerase chain reaction (qRT-PCR) results showed that after treatment with IL-1ra-loaded thermosensitive hydrogel, the levels of inflammatory factors IL-1, IL-6, and TNF-α decreased. Additionally, in diabetic periodontitis rats, it decreased alveolar bone absorption and prevented periodontal inflammation (Liu, Liu, Wang, Zhang, Qu, Liang, Si and Wang, 2022). IL-12 promotes the development of human periodontal ligaments as well as the elevation of RANKL, which leads to alveolar bone loss. By controlling localized microbial elimination, IL-12, on the other hand, protects periodontal tissue (Issaranggun Na Ayuthaya et al., 2018). Consequently, controlling the number of cytokines during periodontitis progression is critical, and regulatory modulation *via* targeted therapy is a promising advancement in periodontal treatment. Hygor et al. discovered that cashew gum-containing Orabase hydrogels inhibited the synthesis of COX-2, NOS-2, INF-α, and MYD88. In rats with periodontitis, increased transcription of anti-inflammation genes (IL-10, IL-4, and TGF1) decreases inflammation (Ferreira-Fernandes et al., 2019).

Other biological products, including ROS, impact the emergence of periodontitis along with cytokines. As a result, regulating the number of cytokines during periodontitis progression is crucial, and regulation manipulation performed by targeted therapy is a prospective development in periodontal treatment (Ebersole et al., 2013). Yu et al. developed cerium oxide nanoparticles that could neutralize ROS inside the cells suppress the MAPK-NF-B signaling route, and lower proinflammatory mediator production. Additionally, in a rat periodontitis model, cerium oxide nanoparticles greatly decreased inflammation and lowered alveolar bone resorption, ultimately promoting tissue regeneration. Cerium oxide nanoparticles have shown promise in periodontal therapy and provided essential knowledge into the utilization of nanozymes in inflammatory diseases. Finally, by controlling cytokines, creative hydrogel delivery strategies can heal periodontitis (Yu et al., 2022).

Lately, Gopalakrishna and associates produced a locally delivered gel filled with piperine. To develop diverse gel compositions, the amount of deacylated gellan gum crosslinked with poloxamer-407 and sodium tripolyphosphate was changed. For 14 days, the ideal liquid was injected into human patients to evaluate its anti-inflammatory effectiveness. The hydrogel was able to develop under physiological settings, allowing for an efficient residence period of the hydrogel within the defect. Additionally, as compared to the control group, there was a substantial enhancement in clinical parameters (Gopalakrishna et al., 2023). CS thermo-responsive hydrogels loaded with antimicrobial peptides (Nal-P-113) and/or antioxidants (polydopamine nanoparticles, PDNPs) could act as a scavenger activity against DPPH was about 80% and perform antimicrobial activities against *Staphylococcus gordonii*, *Fusobacterium nucleatum*, and *Porphyromonas gingivalis* (Ma et al., 2022).

### 8.3 Wound antiinfection dressings

Bacterial pathogens are attracted readily to persistent injuries. This results in delayed healing. Controlling bacterial infection has traditionally been the emphasis of wound dressing and tissue restoration, limiting the probability of wound infection (Cao et al., 2019). Several antimicrobial agents have been widely used in clinical/preclinical trials for bacterial infection (Nainu et al., 2021). Zang et al., for example, loaded ornidazole into chitosan-based hydrogels, which might suppress *P. gingivalis* development and enhance periodontal wound healing of class III bifurcation defects (Zang et al., 2019). Furthermore, diabetic individuals with high blood glucose levels are prone to bacteria-related infections, which impair wound healing (Tao et al., 2015). By combining curcumin’s antibacterial and anti-inflammatory properties with alginate-based hydrogels, Shah et al. efficiently encouraged the healing and treatment of ulcers (Kumari et al., 2022). In a nutshell, limiting bacterial infection with polysaccharide-based hydrogels is the most prevalent technique for reducing the prevalence of wound infection in clinical/preclinical studies.

Numerous commercialized surgical dressings, spanning from organic substances to artificial ones in a range of morphological forms, have been manufactured at this point. Investigators were required to explore innovative wound dressings that utilized natural hydrogels with antimicrobial properties due to antibiotic resistance.

### 8.4 Promotion of periodontal regeneration

The primary reason for periodontal tissue loss, which eventually results in tooth loss, is a decrease in periodontal regeneration ability. The goal of treatment for periodontitis is to stop the deterioration of periodontium while encouraging regeneration and repair of periodontal tissues (Swanson et al., 2022). Finally, by speeding periodontal tissue regeneration, thermosensitive hydrogel delivery methods can successfully cure periodontitis (Li Y et al., 2023).

#### 8.4.1 Gene delivery

Gene therapy, which is an approach for fixing faulty genes that cause certain genetic disorders, attempts to treat numerous genetic ailments. A critical stage in gene therapy is the introduction of a suitable therapeutic gene into the cells, which will replace, repair, or control the malfunctioning gene that triggers the disease. However, because DNA is a negatively charged, hydrophilic molecule, it cannot be transported to the nucleus, thus requiring it to travel across the cell’s outer layer, which is both negatively charged and hydrophobic. As a result, genetic transporters (also known as vectors or vehicles) have been produced (Pan et al., 2021). Viruses are the natural way of transporting genes and were the initial transporters used for the transfer of genes (Howarth et al., 2010).

**FIGURE 5:**
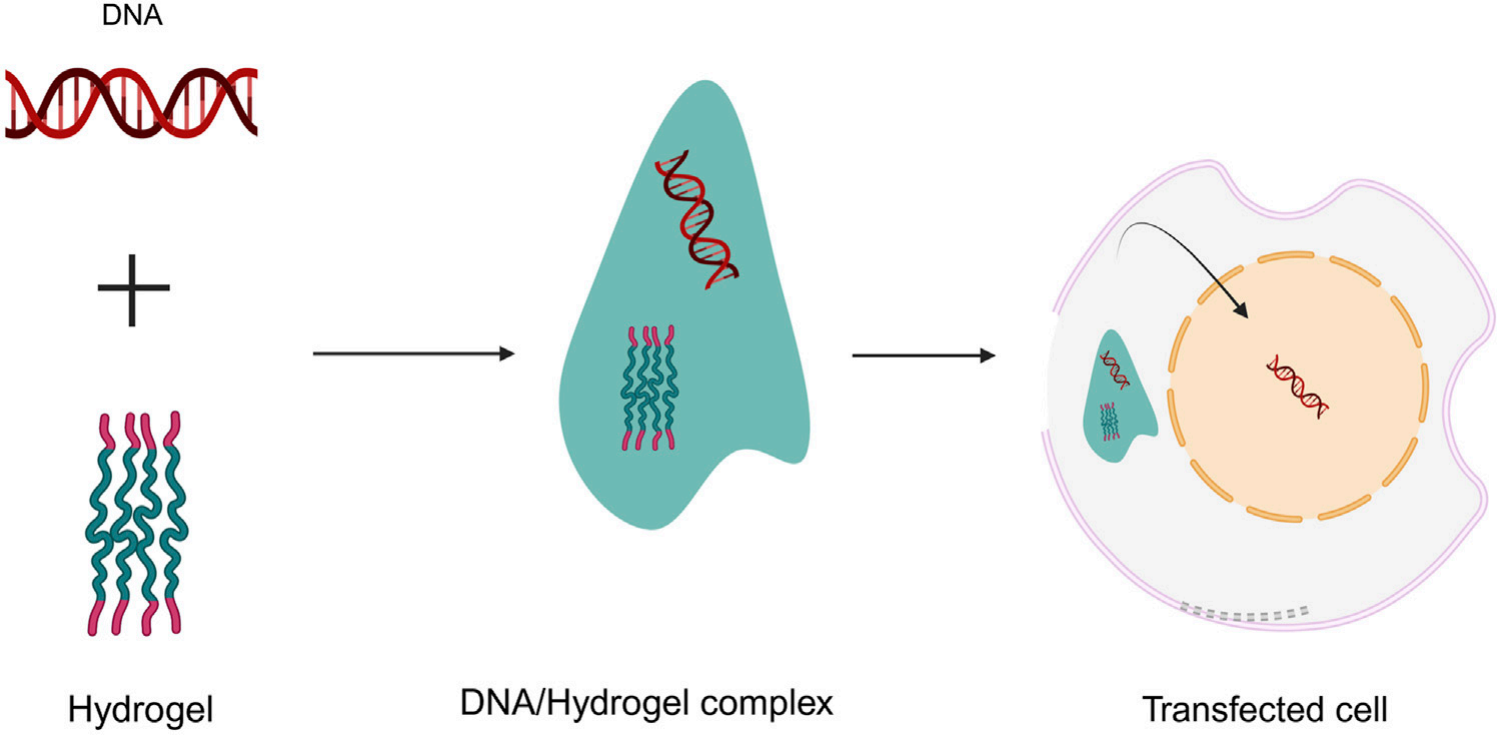
Delivery of genes by polymeric vehicles.

Nevertheless, viruses have several drawbacks, the most serious of which is the immunological response they might elicit, which is why non-viral vehicles have been created (Ramamoorth and Narvekar, 2015). The majority are polymers since they are less costly and safer than viruses, and they are also simpler to adjust than transporters like liposomes. The four main stages of gene delivery when utilizing a polymer carrier are as follows: (1) DNA and polymer complexation; (2) incorporation of the DNA/polymer complex (sometimes called polyplex) onto cells for a duration commonly called transfection time; (3) complex removal from cells; and (4) incubation time, during which the cells are allowed to grow until results are observed (Figure 5). Whereas incubation and transfection are often carried out at 37 °C (the body temperature required for the cells to survive), complexation is typically carried out at room temperature. It is interesting to note that the temperature during complexation, incubation, and/or transfection has been changed using thermo-responsive polymers to boost transfection efficiency (Ward and Georgiou, 2011).

As a periodontium therapy, Li et al. created a composite made up of CS NPs loaded with BMP-2 plasmid DNA (pDNA-BMP2) in CS/β-GP hydrogel. CS NPs protected pDNA-BMP2 against nuclease degradation. Encapsulated CSNPs-pDNA-BMP2 was dispersed with 80% effectiveness throughout the scaffold’s interconnecting pores. It showed outstanding biocompatibility desired cellular development, and multiplication in human periodontal ligament cells, suggesting that it might be used in gene delivery and periodontal repair (Li et al., 2017). Lan et al. created an amalgamated hydrogel dressing capable of detecting and administering MMP-9 siRNA (siMMP-9). SiMMP-9 was mixed with Gly-TETA (GT), and the resulting framework was placed into a thermosensitive hydrogel composed of pluronic F-127 (PF) and methylcellulose (MC) (Lan et al., 2021). Considering its precise mechanism of activity and capacity to swiftly quiet specific expression of genes, siRNA has significant therapeutic promise. The hydrogel distributed GT/siMMP9 into the damaged tissue *via* temperature-dependent control for 7 days, offering regional and constant delivery, which ended in substantial suppression of MMP-9 and an enhancement in the healing process of injuries.

#### 8.4.2 Cell-based therapy

**FIGURE 6:**
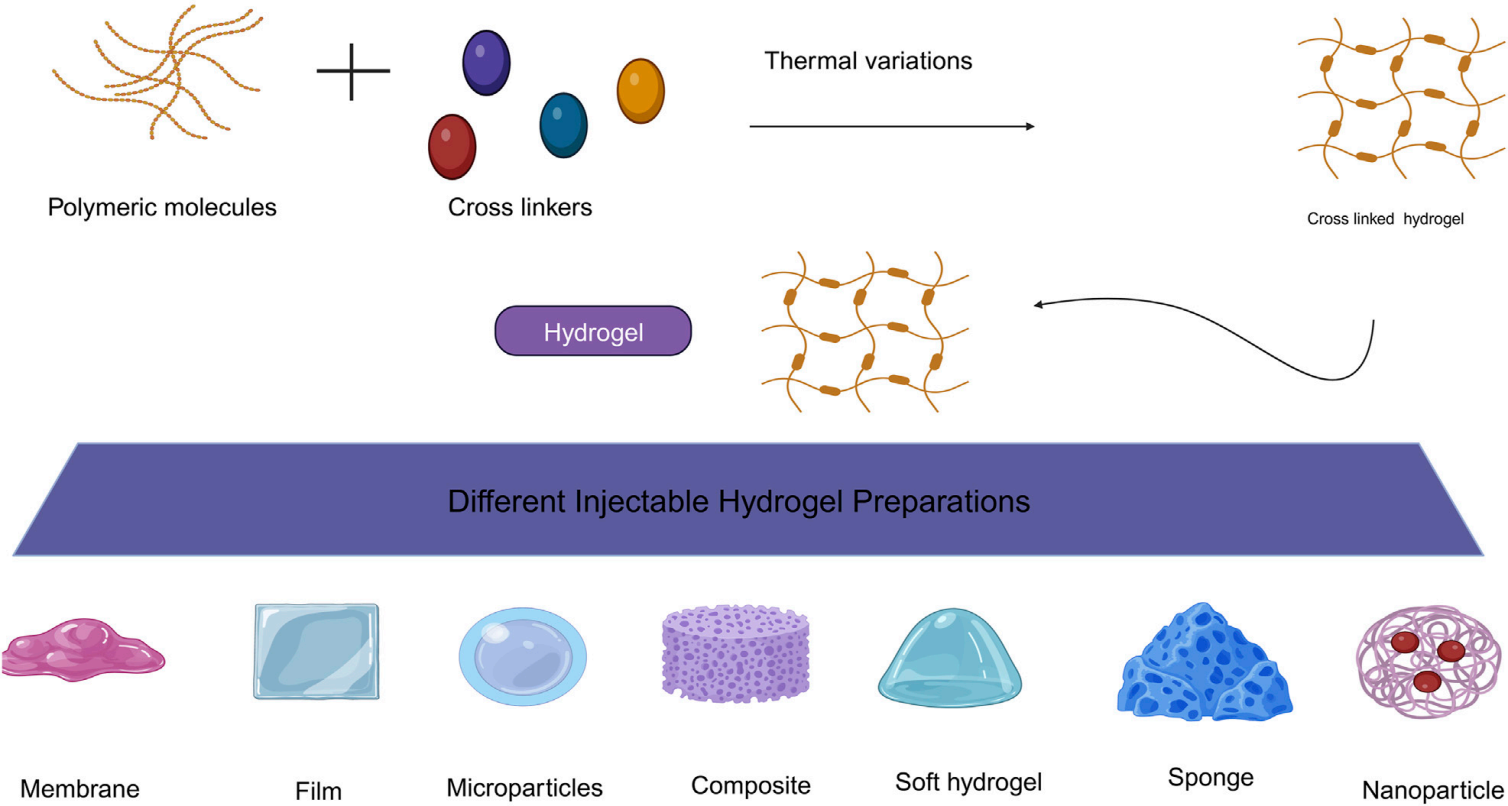
*In situ* formation of a scaffold in tissue engineering.

Cell-based treatment and tissue engineering are the most appealing and frequently researched regenerative medicine options. The direct administration of cells leads to inadequate control, numerous cells lost owing to injection site reflux, and inadequate adhesion into host tissues (Mahla, 2016). Because of their advantages, injectable *in situ* forming thermos-responsive hydrogels are utilized as carriers and transient supportive platforms for cellular and fresh tissue ingrowth (Figure 6; Table 4) (Dimatteo et al., 2018).

**TABLE 4:** Application of injectable thermosensitive hydrogels to deliver cells.

Hydrogel	Delivered cells	Outcomes	References
Cs/β-GP	Dental pulp stem cell-derived exosomes	Encouraging macrophage transformation from a pro-inflammatory to an anti-inflammation behavior	Shen et al. (2020)
CS/collagen/BGP	dog-BMSCs	Osteogenesis, and osteoinduction	Sun et al. (2011)
PGDF-BB/BMP- 2/CS/BGP	PGDF-BB	Continuous discharge of PGDF-BB and BMP-2	Min et al. (2019b)
	BMP-2		
CS/BGP/HAP	DPSCs	Increased osteogenic activity	Chen et al. (2016)
CS/nano-HAP/Na2CO3	rat BMSCs	Improved angiogenesis and osteoinduction	Li et al. (2014)
CS-HTCC/BGP	HPDLCs	Enhanced cellular viability, and regeneration capacity	Ji et al. (2010)
CS-PA/BGP		Improve periodontal tissue regeneration	Zang et al. (2014)
CS/acid-soluble collagen/α-GP/BGP	L929 cells	Offered microenvironment for tissue engineering	Dang et al. (2017)

Bone marrow stem cells (BMSCs), platelet-derived growth factor-BB (PGDF-BB), bone morphogenic protein-2, (BMP-2), chitosan (CS), hyaluronic acid (HA), poly (N-isopropylacrylamide) (PNIPAm), beta-glycerophosphate (BGP), hydroxyapatite (HAP), dental pulp stem cells (DPSCs), Na2CO3, N-hydroxypropyl-3-trimethylammonium chloride chitosan (HTCC), human dental pulp stem cells (hDPSCs), phytic acid (Pa), human osteosarcoma cells (MG-63), glyoxal (Gx).

#### 8.4.3 Growth factors delivery

**FIGURE 7:**
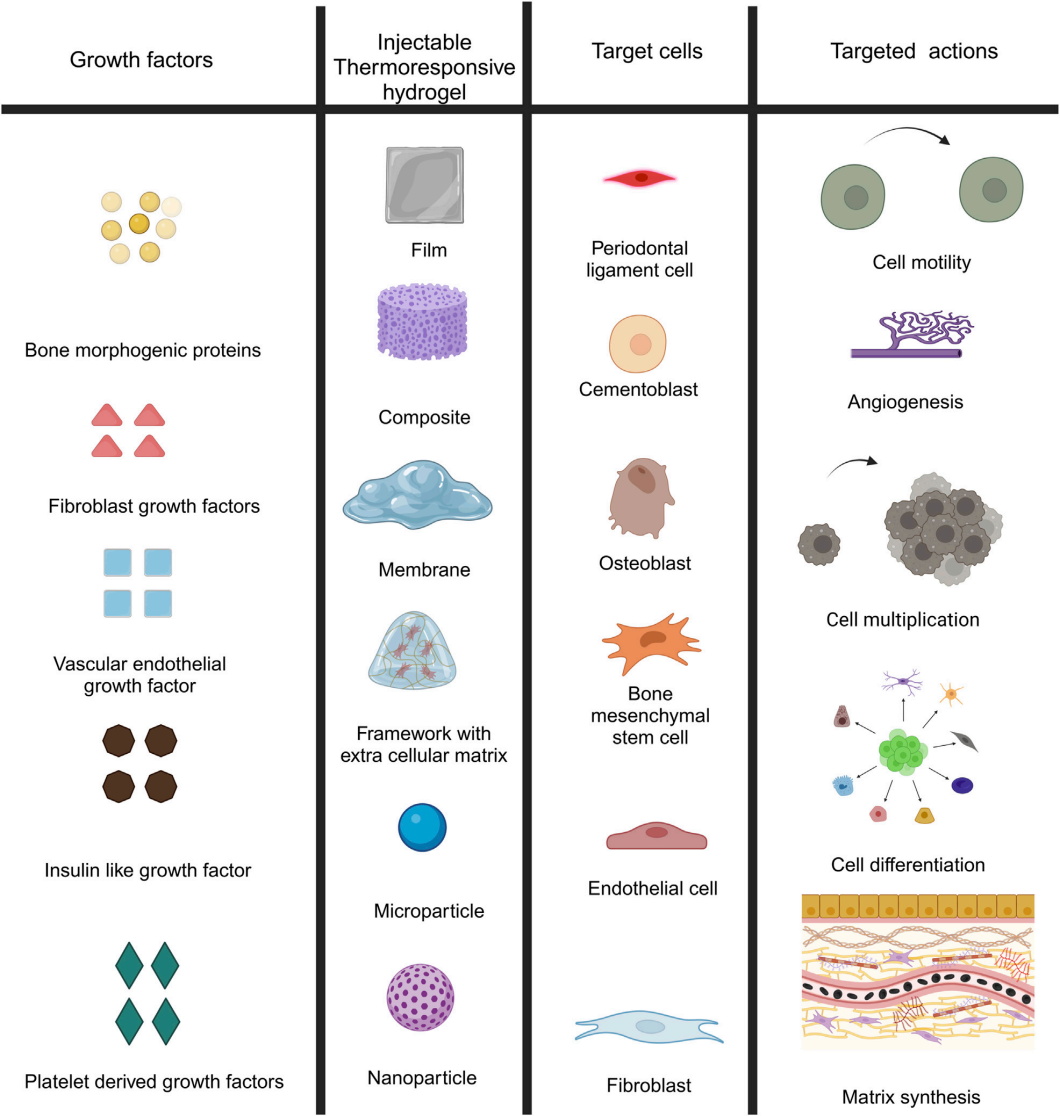
Local application of growth factors *via* injectable thermosensitive hydrogels.

Growth factors assisted delivery via utilization of injectable thermpsensitive natural polymeric hydrogels provide viable, economic, safe, and easy procedure for promotion of periodontal regeneration (Figure 7; Table 5). Periodontal problems were treated with human fibroblast growth factor 2 containing hydrogels. Following 1 year of therapy, the clinical markers of periodontal wound healing improved dramatically in 30 patients (Matsumoto, 2018). Tamura et al. investigated the therapeutic benefits of prolonged release of basic fibroblast growth factor (bFGF) from gelatin hydrogels in patients with periodontal disease and bone abnormalities in separate research (Matsumoto, 2018). Clinical metrics including probing pocket depth decrease, clinical attachment gain, and radiographic bone fill improved significantly 1 year following therapy. Furthermore, no negative consequences were found (Matsumoto, 2018). An injectable chitosan/gelatin thermoresponsive hydrogel loaded with BMP-2 as an osteoinductive biomolecule and T8IC nanoparticles as a photosensitizer was created to improve PDT and BMP-2 release. It demonstrated outstanding bactericidal activity, osteogenic induction, and biosafety *in vitro* and *in vivo*. Furthermore, immunohistochemical staining and micro-CT scans indicated that PTT and PDT might enhance bone repair by reducing inflammation (Wang et al., 2023).

**TABLE 5:** Application of injectable thermosensitive hydrogels to deliver growth factors.

Hydrogel	Bioactive agent	Model		Outcomes	References
SDF-1/chitosan/carboxy methyl-chitosan nanoparticles (SDF-1/CS/CMCS NPs)	stromal cell-derived factor-1	rat calvaria defects		Cumulative release of SDF-1 was only 40%. Stimulate new bone formation	Mi et al. (2017)
Chitosan/gelatin-based hydrogel	erythropoietin	maxillary sinus floor defect in rabbit		EPO significantly boosts BMSC proliferation	Li D et al. (2020)
Collagen/Alginate	nHAP, hNGFβ	Rabbit mandibular distraction osteogenesis		HNGF was safe and able to continue its biological activity	Cao et al. (2012)
				Following 14 days, hNGF in COL/nHAp/AG hydrogel improved the reconstruction of bones	
Cs/gelatin/glycerol phosphate hydrogel	BMP-6			*In vitro*, enhance bone formation, and the transformation of PDL development and creation of new connective tissue. *In vivo*: significant mineralization Improved bone quantity, trabecula numbers, and trabecular width, reduced levels of inflammatory cytokines	Chien et al. (2018)
CS/thioglycolic acid/Ti6Al4V	rhBMP-2			Enhance bone regeneration	Chew et al. (2019)
Gel	iPRF	*In vitro*	*In vivo*	GelNPs worked as carriers for continuous discharge of growth factors from iPRF GelNPs-iPRF mixture improved the reconstruction of bones following 8 weeks	Mu et al. (2020)
		HUVECs	Rabbit sinus augmentation		
PGDF-BB/BMP- 2/CS/BGP	PGDF-BB BMP-2			Prolonged discharge of PGDF-BB and BMP-2	Min et al. (2019a)
CHX/β-CD/CS/BMP-2/alginate	chlorhexidine rhBMP-2			Sustained liberation of CHX and rhBMP-2	Ma et al. (2007)
HA	nHAp, BMP-2	Subperiosteal injection in mandibular rat diastema		Bmp-2 levels were shown to be closely connected to bone formation, and revascularization. nHAP, and BMP-2 worked together to boost hydrogel bone forming potential. Following 8 weeks, HA-based hydrogels incorporating nHAP, and BMP-2 augmented mandibular bone	Martínez-Sanz et al. (2012)
Chitosan/β-glycerophosphate	Bone morphogenetic protein 7/ornidazole	Furcation defects in dogs		Enhanced periodontium regeneration. Inhibited *Porphyromonas gingivalis* growth	Zang et al. (2019)

Stromal cell-derived factor-1(SDF-1), chitosan (CS), carboxymeymethy-chitosan nanoparticles (CMCS NPs), PCL, BMP-2 BMSCs, Fmoc-Phe3 hydrogel (FPH), Reactive oxygen species (ROS), bone morphogenic protein-6 (BMP-6), Recombinant bone morphogenic protein-2 (rhBMP-2), Chlorhexidine (CHX), Beta cyclodextrin (β-CD), Poly (N-isopropylacrylamide) (PNIPAM05), platelet rich plasma (PRP), infrapatellar fat pad stem cells (IFPSC), glycosaminglycan (GAGs), Alkaline phosphatase (ALP), Beta tricalcium phosphate (BCP), rat adipose stem cells (rADSCs), human nerve growth factor beta-hNGFβ), nanohydroxyapatite: nHAP.

#### 8.4.4 Local drug delivery

As the name implies, drug delivery is the approach or procedure of giving a medication molecule (drug) to create a medicinal benefit. The delivery of the medicine to the appropriate place, at a suitable t time, and the effective concentration are critical. Nevertheless, there are several barriers to effective medication delivery. These challenges include the medications’ poor solubility, environmental or enzymatic degradation, rapid levels of excretion, general harmful effects, and difficulty in traversing biological barriers, to mention a few. To solve these challenges, drug delivery vehicles, the majority of which are founded on polymers, are being employed. Nonetheless, even when employing a carrier to transport the medicine, achieving the desired regulated release rate is challenging (Atia et al., 2022a).

The distribution of medicine is frequently troublesome since the level of the released drug at the target location is either excessively low or elevated, and it is not administered for the desired period. As a result, “smart” polymeric carriers are being employed to transport pharmaceuticals. By just dispensing the medication in response to stimulation from the outside, these delivery systems ensure that the medication gets delivered at an appropriate moment and in sufficient quantities (Table 6). For instance, as the temperature rises, the polymer chains of a vehicle could stretch, allowing the medicine to diffuse out and be liberated from the carrier (Alkhursani et al., 2022b).

**TABLE 6:** Application of injectable thermosensitive hydrogels to deliver drugs.

Hydrogel	Bioactive agent	Model	Outcomes	References
Sodium alginate/methylcellulose/poloxamer 407	Dried Scutellaria baicalensis extract/CS	*In vitro*	Improved drug bioactivity, and antimicrobial capacity	Chanaj-Kaczmarek et al. (2021)
Thermosensitive and mucoadhesive chitosan *in situ* gels	Moxifloxacin		The hydrogel released moxifloxacin for 8 h and had a remarkable antibacterial activity against S*treptococcus* mutans, and *Aggregatibacter Actinomycetemcomitans*	Sheshala et al. (2019)
Thermoresponsive alendronate sodium/PLGA nanoparticles hydrogel	Alendronate sodium	0.5 × 0.5 cm critical size defect in tibia of New Zealand female rabbits	Enhanced fibroblasts formation	Öz et al. (2020)
			Enhanced bone formation	
CS films, and microparticles	Metronidazole		Cs microparticles including hydrogels outperformed the films in terms of their therapeutic profiles	Pichayakorn and Boonme, (2013)
Thermoresponsive Pluronic® F-127 (PF-127)/Hydroxy Ethyl Cellulose (HEC) hydrogel	Azithromycin dihydrate		Hydrogels dramatically influenced regulated azithromycin release *in vitro*	Venkatesh et al. (2013)
			Considerable enhancement in periodontitis clinical variables	
Injectable gelatin alginate hydrogels	Metronidazole		The metronidazole-laden hydrogels demonstrated long-lasting dissolution, with the majority of the loaded metronidazole eluting within 24 h. Following 24 and 48 h, *in vitro* fibroblast vitality was at least 75% (when tested using human fibroblasts), showing good biocompatibility	Zussman and Zilberman, (2022)
Alginate-based *in situ* gels	Doxycycline HCl		Sodium alginate and hydroxypropyl methylcellulose were used to make this product. A continuous doxycycline delivery rhythm lasting more than 12 days	Obaidat et al. (2010)
Mucoadhesive cellulose gel	Metronidazole		Metronidazole extended continuous release *in vitro*	Sallam et al. (2015)
Chitosan/sodium β-glycerophosphate/gelatin	Aspirin/erythropoietin	Rat	Sustained drugs release	Xu et al. (2019)
			Anti-inflammation, and periodontal regeneration	

Polycaprolactone nanoparticles (PCL NPs), chitosan (CS), silk fibroin (SF), poly (N-isopropylacrylamide) (PNIPAM), oligoproline (PRO).

Gad and coworkers developed solid lipid microparticle gels containing doxycycline hydrochloride and metronidazole and demonstrated their therapeutic usefulness in periodontitis (Gad et al., 2017). Meloxicam (Mex)-loaded-CD ISG and ISM were created by combining -CD in dimethyl sulphoxide (ISG) as the internal phase and camellia oil containing 5% glyceryl monostearate (ISM). A 24-gauge needle was used to conveniently inject Mex-loaded -CD systems with 40% CD. Following Fickian diffusion, the ISM system containing 40% -CD converted into microparticles and prolonged the drug release to 7 days with a reduced initial burst release.

Furthermore, the considerable weight loss indicated possible degradability. The sluggish diffusion rate of the solvent from the ISM system was triggered by a high maximum deformation force with a highly viscous nature. As a result, 40% CD ISM is a viable anti-inflammatory medication local Mex-controlled release mechanism for periodontitis therapy (Rein et al., 2020).

#### 8.4.5 Delivery of other biological molecules

**FIGURE 8:**
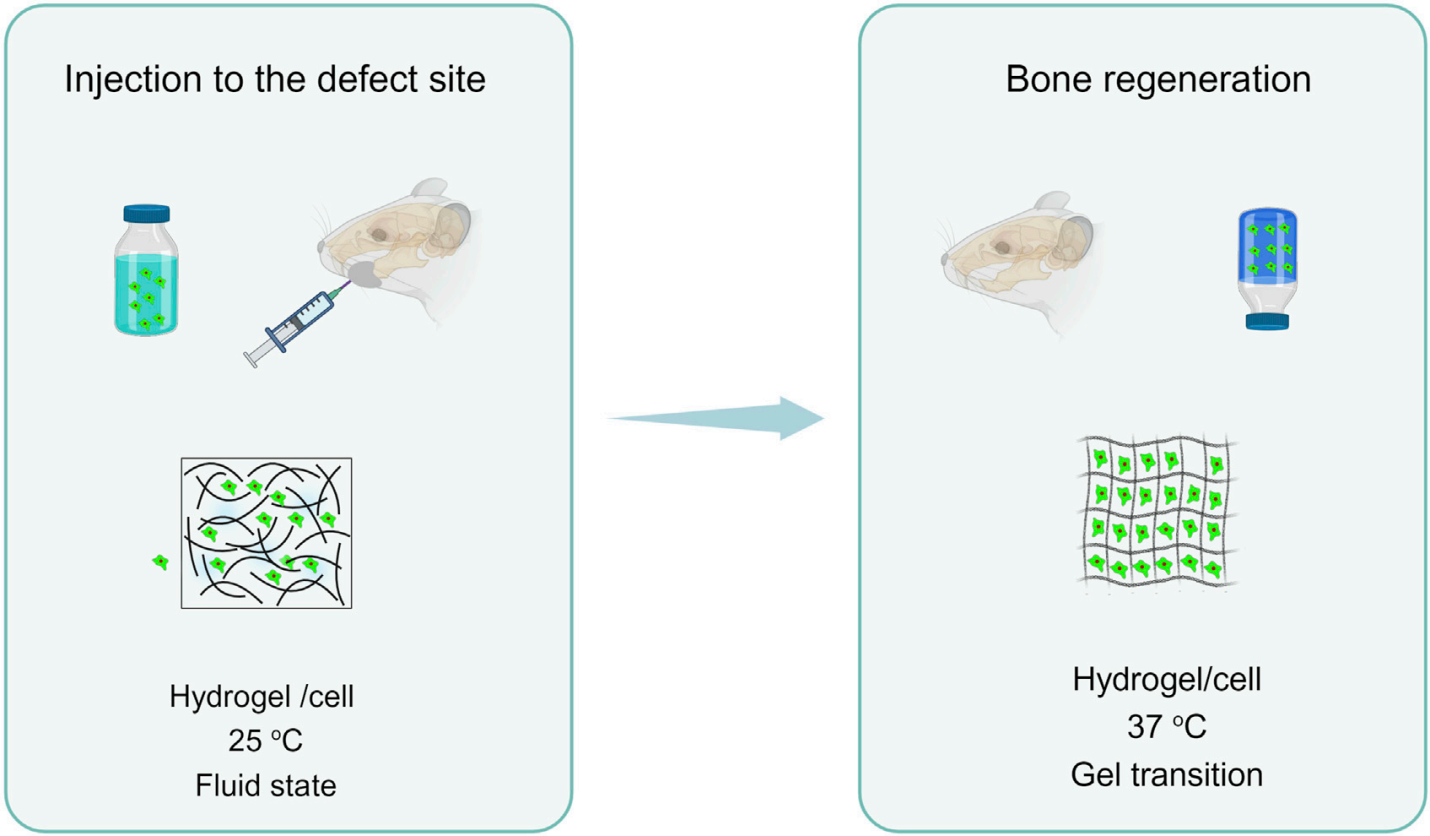
*In situ* formation of a scaffold in bone tissue engineering.

The injectable hydrogels are naturally adaptable, allowing them to fill irregular-sized lesions, and a minimally invasive method assists in the administration of bioactive substances and cells (Figure 8; Table 7) (Dhivya et al., 2015). β-TCP-loaded thermosensitive chitosan hydrogels showed biocompatibility with MC3T3-E1 and HGF cells. For 14 days (Tan et al., 2023). To be rigid enough for tissue regeneration, a biocompatible grafting polymer-based temperature-sensitive hybrid hydrogel (Chitosan-P123, CP) comprising gelatin and curcumin could sustainably release curcumin, as well as a superior biocompatibility scaffold (Pham et al., 2019). The potential of particularly generated pharmaceutical release at the wound bed was demonstrated by encapsulating curcumin nanoparticles with MMP9-responsive and thermos-sensitive gelatin hydrogel, which increased the efficiency in healing the standardized skin wounds in streptozotocin-induced diabetic mice (Liu J et al., 2018). Lszewska-Czyz and others revealed that using hyaluronic acid hydrogels as an addition to non-surgical periodontal treatment resulted in better clinical outcomes after 3 months (Olszewska-Czyz et al., 2021). Probing depth reduction was unaffected, however there was an increase in periodontal attachment (1 mm more than the control group) and a decrease in inflammation, as measured by bleeding on probing (6% less than the control group). Moreover, no adverse consequences have been documented (Olszewska-Czyzet al., 2021).

**TABLE 7:** Application of injectable thermosensitive hydrogels to other biological molecules.

Hydrogel	Bioactive agent	Model		Outcomes	References
Zinc-doped CS/nano-HAP/BGP	nano-HAP			MSC multiplication Bone repair *via* collagen and apatite accumulation	Dhivya et al. (2015)
Cs/silk fibroin/hydroxyapatite/GP hydrogels	hydroxyapatite			Desirable degradation pattern	Wu et al. (2016)
CS-PA/BGP				Enhanced periodontium regeneration	Zang et al. (2014)
CS/HA	nHAp	*In vitro*	*In vivo*	The hydrogel offered a 3D platform for stem cell development, division, and transformation	Pan et al. (2020)
		ME3T3	Rat alveolar bone defect (tooth extraction)	The breakdown of loaded nHAp created an elevated level of calcium and phosphorus, which encouraged stem cell osteogenic development. Following 4 weeks, the hydrogel-nHAp composite scaffold revealed faster alveolar ridge preservation	
CS/BGP/nanoHAP/collagen	nano-HAP			Growing MSCs	Huang et al. (2009)
PNIPAM/Starch/CS				Enhance the cytocompatibility and degradation of the scaffolding	Vieira et al. (2014)
CS/N-acetylcysteine/PNIPAM				There is no toxicity to fibroblasts, MSCs, or osteoblasts	Wu et al. (2018)
HA/CS/PNIPAM/BCP	BCP			Encourage bone formation Osteoblastic genetic transcription	Chen et al. (2013)
nano-HAP/CS/CNTs	BSA and OVA			BSA and OVA were released gradually	Cancian et al. (2016)
Zinc-doped CS/nano-HAP/BGP	nano-HAP			MSC multiplication Bone repair *via* collagen and apatite buildup	Dhivya et al. (2015)
CS/collagen	Mineralized collagen			Tissue engineering	Huang et al. (2011)
BGNPs encapsulated in CS-BGP/BGNPs/CS/collagen	Bioactive glass nanoparticles (BGNPs)			Increased production of bone-like apatite	Couto et al. (2009)
CS/collagen/carbon nanotubes (COOH-SWCNTs)	carbon nanotubes (COOH-SWCNTs)			Enhanced porous structure	Kaur et al. (2021)
				Enhanced bioactivity	
				Boosted osteoblasts division, and quick deposition of hydroxyapatite	

BGP, Phytic acid (PA), collagen, Poly (N-isopropylacrylamide) (PNIPAM), beta tricalcium phosphate (BCP), nanohydroxyapatite (nano-HAP), carbon nanotubes (CNTs), bovine serum albumin (BSA), ovalbumin (OVA), bioactive glass nanoparticles (BGNPs).

## 9 Concluding remarks

The creation of innovative stimulus responses determined by sophisticated materials and preparation processes is critical to smart hydrogel design. Furthermore, ongoing in-depth research on the pathogenic process of periodontitis has yielded several possible therapy methods. Smart hydrogels have produced great therapeutic results to date. With the advancement of stimulus-reactive techniques and the development of stronger pathogenic mechanisms of periodontitis, there is still an enormous opportunity for the fabrication of innovative intelligent hydrogels for periodontal therapy. We expect that intelligent hydrogels will offer fresh options for periodontitis treatment in the coming years.
